# Improvement of the Champagne–Epernay–Geisenheim (CEG) Wine Yeast via Meiotic Spore Clone Selection

**DOI:** 10.3390/microorganisms14081679

**Published:** 2026-07-31

**Authors:** Judith Muno-Bender, Jennifer Badura, Katrin Matti, Kerstin Zimmer, Silvia Brezina, Heike Semmler, Paul Zeiss, Doris Rauhut, Jürgen Wendland

**Affiliations:** 1 Department of Microbiology and Biochemistry, Hochschule Geisenheim University, von-Lade Strasse 1, 65366 Geisenheim, Germanyjennifer.badura@hs-gm.de (J.B.); doris.rauhut@hs-gm.de (D.R.); 2 Geisenheim Yeast Breeding Center, Hochschule Geisenheim University, von-Lade Strasse 1, 65366 Geisenheim, Germany; 3 Lallemand Deutschland GmbH, Färberstraße 22, 95126 Schwarzenbach an der Saale, Germany

**Keywords:** *Saccharomyces*, wine yeast, hybrid, meiosis, sporulation, heterosis, fermentation, yeast breeding

## Abstract

Climate change is altering grape composition and ripening patterns, thereby influencing the selection criteria for wine yeast strains adapted to warmer conditions and fluctuating sugar–acid balances. Previously, we sequenced and annotated the Champagne–Epernay–Geisenheim white wine yeast CEG. CEG is a hybrid tetraploid strain with more than three genome equivalents of *Saccharomyces cerevisiae* and only 1/3 of a *S. kudriavzevii* genome. To address climate change issues, we used the limited sporulation capacity of CEG to isolate meiotic progeny. The obtained spore clones were subjected to targeted selection procedures, identifying progeny with enhanced fermentation performance under higher temperature conditions. Spore clones were also selected to carry the *FOT4* allele encoding a Fungal Oligopeptide Transporter gene. We identified suitable strains, particularly GYBC 1467, that surpassed the parental strain at 25 °C with enhanced volatile aroma production. Genome sequencing of three spore clones indicated differential loss of *S. kudriavzevii* DNA. This approach demonstrates that, via a single round meiosis, spore clone isolates with substantially improved fermentation performance compared with the parental strain can be obtained. This provides a fast route for the generation of resilient wine yeasts capable of maintaining wine quality in increasingly difficult fermentation environments.

## 1. Introduction

Rising global temperatures, altered precipitation patterns, and increased weather variability are fundamentally reshaping viticulture worldwide [1,2,3]. These shifts advance grape ripening, alter vine phenology, and yield musts with elevated sugar (>220 g/L), reduced acidity, softer tannins, and diminished aroma precursors [1]. High sugar drives alcohol levels beyond 14% vol, while low acid levels impair freshness, balance, and aging stability. Heat stress further curbs varietal aromas like monoterpenes (geraniol, linalool) and norisoprenoids (β-damascenone) [4].

Fermentation temperatures also rise, altering ester (acetate/ethyl) and higher alcohol (isoamyl alcohol, 2-phenylethanol) profiles—reducing fruity notes via hydrolysis and boosting fusel oils—yet offering aroma modulation potential [5,6,7]. Ethyl esters (e.g., hexanoate, octanoate) form best at lower temperatures, while acetate esters (e.g., isoamyl acetate) increase with heat [8].

These changes in must composition challenge *Saccharomyces cerevisiae* wine strains domesticated over centuries for temperate-climate musts. They now face osmotic stress and low nitrogen-induced sluggishness, increasing the risk of acetate formation and stuck fermentations.

Indigenous non-*Saccharomyces* yeasts (e.g., *Hanseniaspora, Starmerella*) dominate early fermentation phases. *Hanseniaspora*’s success is attributed to its rapid growth rate, potentially enabled by ancestral gene losses and ongoing adaptive evolution to the grape and fermentation niche [9,10,11].

*Saccharomyces cerevisiae* has been associated with human activities throughout the ages as a Kulturfolger and then as our first domesticated species [12]. Commercial dry yeasts ensure reproducible fermentations; however, these starter cultures are extremely similar genetically, suggesting domestication bottlenecks [13]. Many popular strains (e.g., VIN7, CEG) are *S. cerevisiae* × *S. kudriavzevii* hybrids [14,15,16,17]. Several non-*Saccharomyces* starter cultures, including *H. vineae*, *Lachancea thermotolerans*, *Metschnikowia pulcherrima*, *Torulaspora delbrueckii*, now complement them for diversity [18,19].

Consumer resistance to genetically engineered yeasts has positioned classical breeding and selection as the primary tools for developing climate-adapted strains [20]. These non-GMO approaches—ranging from sexual hybridization and spore-to-spore mating [21,22] to adaptive laboratory evolution (ALE) and high-throughput screening [23,24]—enable predictable fermentation performance superior to co-inoculation strategies.

Yeast breeding, however, is often limited in domesticated strains due to their hybrid state, aneuploidies or polyploidy. Triploid wine yeast strains like VIN7, group I lager yeast strains of the *Saaz* type, e.g., *S. carlsbergensis* or other non-sporulating strains, are therefore not amenable to improvement by yeast breeding. Breeding of interspecific *Saccharomyces* hybrids has been successfully used to select strains with improved osmotolerance, low-temperature tolerance, or improved fermentation rates [25]. However, these hybrids suffer from postzygotic sterility barriers and are thus difficult to subject to further rounds of improvement [26]. The wine yeast Champagne–Epernay–Geisenheim, CEG, is a chimera/hybrid between *S. cerevisiae* and *S. kudriavzevii* [14]. As has been shown with other *S. cerevisiae*/*S. kudriavzevii* hybrids, there has been a reduction in the *S. kudriavzevii* genome in CEG, which holds 3 2/3 genomes of *S. cerevisiae* but only 1/3 of the *S. kudriavzevii* genome, with a total of ~3.85 Mb *S. kudriavzevii* DNA [14,27]. CEG is known to ferment slowly and can run into stuck fermentations in stressful conditions at low temperatures or low N supply. Remarkably, CEG retains sporulation capacity, creating opportunities for targeted strain improvement. This rare sporulation phenotype in the CEG hybrid offers a unique opportunity for rapid adaptation. Meiosis-driven reassortment enables segregation of suboptimal alleles (e.g., cold adapted *S. kudriavzevii* respiratory genes) while preserving adaptive genomic blocks (e.g., *S. cerevisiae* fermentation genes). Unlike mutagenesis, which introduces random lesions, or protoplast fusion, which often yields unstable polyploids, sporulation harnesses the natural recombination potential of hybrid genomes to generate genetic diversity.

The aim of this study was, therefore, to exploit the sporulation capacity of CEG to isolate and characterize spore clone-derived strains. Using a combined screening strategy based on plate assays and fermentation tests, we identified superior isolates that outperformed the parental CEG strain in key fermentation traits at higher fermentation temperatures. Whole-genome sequencing further revealed losses of *S. kudriavzevii* chromosomal material in these evolved strains. More broadly, this approach may be extended to other wine yeast strains as a route to quickly selecting variants with greater resilience to climate change-driven shifts in grape must composition and fermentation conditions.

## 2. Materials and Methods

### 2.1. Strains, Media, and Fermentation Setup

The Champagne–Epernay–Geisenheim/Epernay 2 (CEG, GYBC 141) wine yeast strain used in this study was obtained from the Geisenheim Yeast Breeding Center (GYBC) culture collection. Strains were propagated in YPD (10 g/L yeast extract, 20 g/L peptone, 20 g/L dextrose) at 30 °C. Plate assays were performed using YPD plates at 10 °C, 30 °C, and 37 °C, or on YPD plates supplemented with 1 M NaCl at 30 °C. For the plate assays, cultures were grown overnight in YPD and 2 µL per culture was spotted. Plates were photographed after two days and six days. Fermentations were inoculated at 10^6^ cells/mL in Riesling must obtained from the university’s winery and fermented at either 10 °C or 25 °C. The same Riesling must batch (HGU vinery, 2024 vintage) was used for all fermentations in this study: Must with Brix 17.93°, 101.5 mg/L yeast available nitrogen—64.4 mg/L were free amino nitrogen (FAN) and 37.1 mg/L ammonium was used that contained 83.6 g/L glucose and 83.8 g/L fructose (167.4 g/L total sugar), pH 3.0, with 6.2 g/L tartaric acid, and 5.3 g/L malic acid. Nitrogen content was determined as described previously [14]. Fermentation progress was monitored using an ANKOM Rf Gas Production system (ANKOM Gesellschaft für Analysentechnik-HLS, Salzwedel, Germany). Stirred fermentations (300 rpm) were run with a volume of 150 mL at 25 °C in a modified 250 mL bottle carrying an Rf sensor module of the ANKOM system.

### 2.2. Sporulation and Tetrad Dissection

CEG was inoculated in liquid YPD and grown overnight at 25 °C. The preculture was centrifuged and washed with H_2_O and finally resuspended in 1 mL H_2_O. Of these precultures, 100 µl were spread on each sporulation plate (2% potassium acetate, 0.22% yeast extract, 0.05% glucose, 0.08% Complete Supplement Mixture (CSM) supplemented with 20 μg/mL adenine and adjusted to pH 7). Sporulation was monitored microscopically. Tetrad dissection was performed using a micromanipulator (MSM 400, Singer Instruments, Roadwater, UK) to generate spore clones. Only asci with four spores were dissected. Germination and colony forming efficiency was quantified after 10 days of incubation at 25 °C.

### 2.3. Phenotypic Mating-Assay

Spore clones may retain the ability to mate with strains of opposite mating type. To test for this mating ability, a plate halo assay was employed, as described in [28]. To this end, a lawn of cells of pheromone sensitive tester strains, either G213 (*MATa*, *bar1::YES1*) or G215 (*MATα*, *sst2::YES1*), was plated on YPD plates. Cells of query strains (2 μL of each of the strains, corresponding to ~ 1 × 10^6^ cells), were spotted on top of the lawn. The plates were then incubated overnight at 30 °C, imaged, and assayed for halo formation. A positive interaction results in growth arrest in cells of pheromone sensitive tester strains of the opposite mating type, leading to a halo of growth inhibition around the query strain colonies.

### 2.4. Amplification of DNA-Fragments

The presence of the *FOT4* gene was assessed by PCR with primers #1498: GATGGCCTGTATGCTGTTCAG and #1499: CCAGGCACGATGGCGTATT. Mating type was determined with primers #25-*MATα*: GCACGGAATATGGGACTACTTCG, #26-*MATa*: ACTCCACTTCAAGTAAGAGTTTG, and #27-*MAT*-locus: AGTCACATCAAGATCGTTTATGG, as described previously [29]. PCR reactions were carried out using standard conditions.

### 2.5. Analytical Methods

Amounts of glucose and fructose, organic acids, and ethanol were analyzed in musts and at the end of fermentations by high-performance liquid chromatography (HPLC). Aroma compounds (higher alcohols, fatty acids, and derived esters) were analyzed by headspace solid-phase microextraction gas chromatography–mass spectrometry (HS-SPME-GC-MS) using an Agilent GC 7890A (Agilent, Santa Clara, CA, USA) system coupled to an MSD 5977B mass spectrometer (Agilent, Santa Clara, CA, USA), as described previously [30]. Nitrogen content was analyzed by determining the primary amino acid content (FAN) using the o-phthaldialdehyde (NOPA) method [31], and by measuring ammonium using the Rapid Ammonium kit from Megazyme (Bray, Ireland).

### 2.6. Beta-Glucosidase Activity Assay

Activity of β-glucosidase was tested in a qualitative liquid assay by monitoring the hydrolysis of esculin (6,7-Dihydroxycumarin-6-glycosid, Carl Roth GmbH, Karlsruhe, Germany) to esculetin, which forms a black complex with Fe(III)-ions. Liquid esculin medium was prepared containing 1 g/L esculin, 0.3 g/L FeCl_3_ 6H_2_O, 25 g/L yeast extract, 1 g/L peptone, and 8 mL glycerol (pH 6). Precultures of all strains were grown overnight in YPD. Cell concentrations were determined and cultures were initially diluted to 1 × 10^7^ cells/mL. Thereafter, ten-fold serial dilutions were generated, and 100 µL of cell suspension was used to inoculate 400 µL of esculin medium in multiwell plates. Plates were incubated at 25 °C and images were acquired after two and three days.

### 2.7. Fermentation Kinetics

The cumulative gas pressure at each time point during fermentation was converted into the number of moles of CO_2_ produced according to the ideal gas law, as published previously [32]. Then, the time at which 50% of the maximum CO_2_ production was reached was determined by linear interpolation. The relative growth rate (µrel) was calculated using the secant method [32].

### 2.8. Statistical Analyses

Statistics were carried out on aroma production with triplicate fermentations. GraphPad Prism version 10.3.1 (GraphPad Software, San Diego, CA, USA) and Excel (Microsoft Office Professional Plus 2019, Redmond, WA, USA) were used for generating graphics. The software JASP Team (Version 0.95.4) (computer software) was used for statistical evaluation. A classical ANOVA was employed, including a homogeneity test with Welch’s correction and a post hoc test using the Games–Howell test. The significance level was set at *p* < 0.05 when comparing with the CEG parental strain.

Illumina NextSeq 2000 whole genome sequencing and assembly of reads into contigs was done by Starseq (Mainz, Germany). Accession numbers for BioProject PRJNA941226 are SAMN56543178 (GYBC 1467), SAMN56543179 (GYBC 1468) and SAMN56543180 (GYBC 1471).

## 3. Results

### 3.1. Isolation of CEG Spore Clones

**Figure 1:**
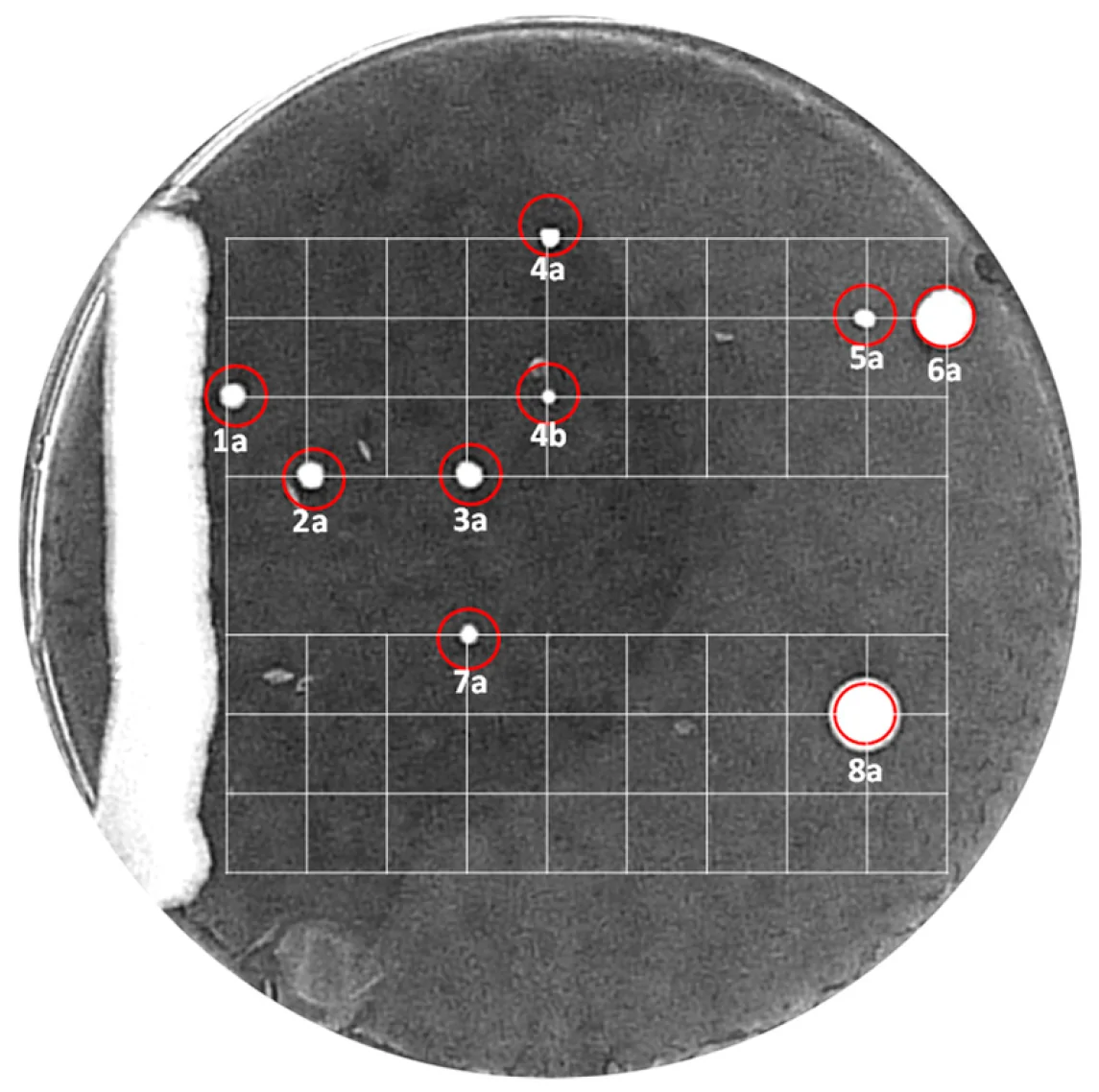
**Microdissection of CEG tetrads**. Plate A is shown (see Table 1). The upper and lower grids indicate the positions of spore deposits from ten tetrads each. Encircled are spore clone colonies. The nomenclature designates a number and letter to each spore clone: the number originates from the tetrad that produced a spore clone, and letters (a to c) are assigned to the spore clone of a single tetrad. In this example, nine spore clones were retrieved. Later spore clones were assigned GYBC strain collection numbers. Throughout the text both numbers are used for convenience.

Genome sequencing of the CEG strain indicated that the ploidy is 4n-1 and the strain is a hybrid between *S. cerevisiae* and *S. kudriavzevii* with a strong bias towards *S. cerevisiae* with more than three genome equivalents [14]. This may explain the sporulation ability we observed in CEG. However, aberrant sporulation was observed that also led to the formation of a small percentage of 8-spored asci. In several rounds we picked and dissected 178 tetrads from different sporulation plates via micromanipulation (Table 1). No viable colonies were formed in half of the tetrads; conversely, none of these tetrads yielded four growing spore clones. In most cases, only one or two colonies could be obtained from a tetrad; only two cases yielded three spore clones (Figure 1).

In total, 124 spore clones from 178 tetrads were formed, corresponding to a germination and successful colony formation frequency of ~17.4%. This distribution reflects extensive failure of *S. cerevisiae/S. kudriavzevii* chromosome pairing during meiosis I prophase, resulting in nondisjunction and spore inviability.

#### 3.1.1. Screening of Spore Clones via Plate Assays

**Figure 2:**
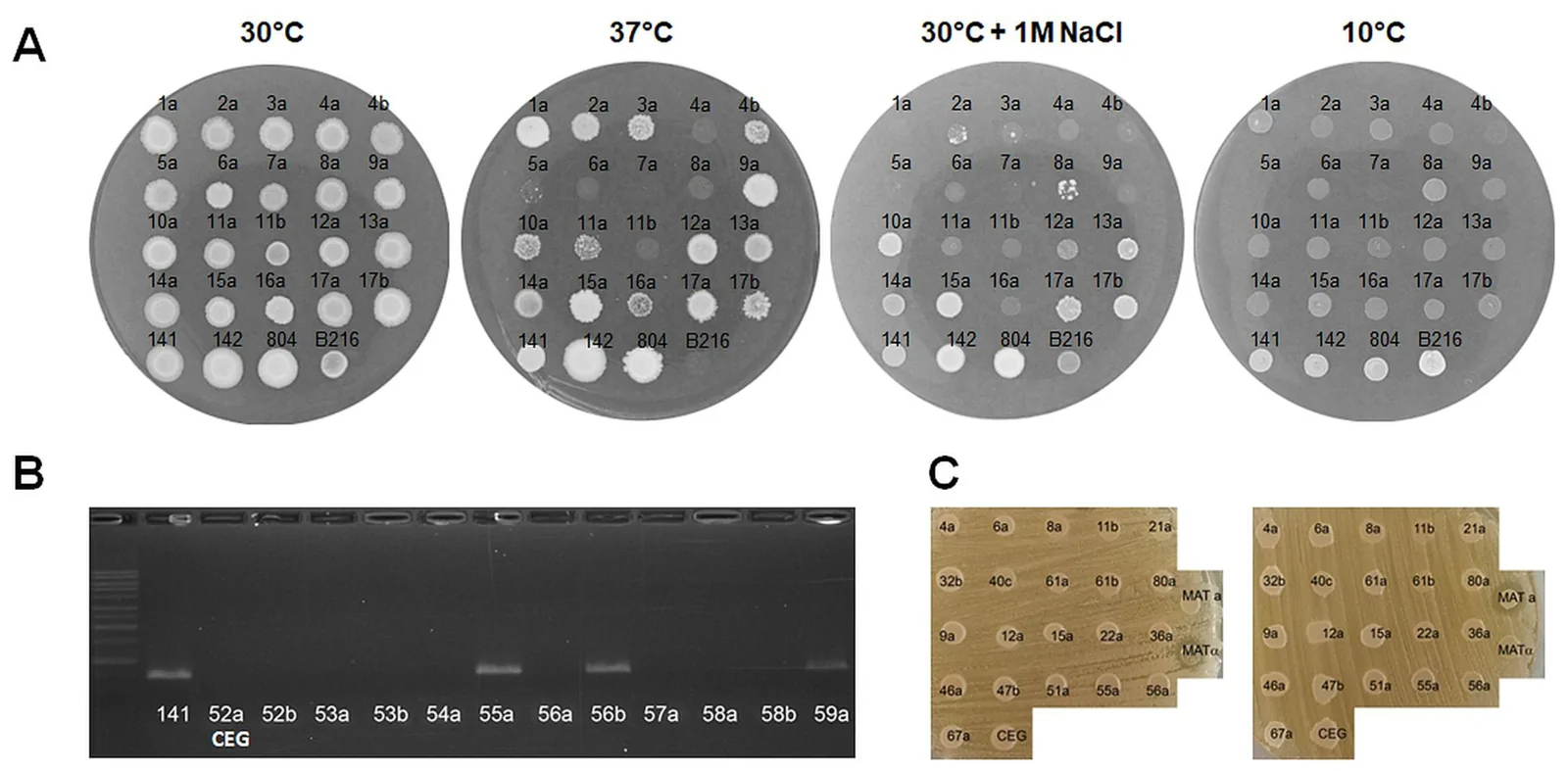
**Characterization of spore clones.** (**A**) Spore clones were spotted on YPD plates, incubated for six days at the indicated temperatures, and then photographed. YPD plates in one set contained 1 M NaCl. The bottom row of each plate contains control strains: 141: CEG; 142: Geisenheim wine yeast GHM; 804: EC1118 wine yeast; and B216: CBS 8840 S. kudriavzevii. (**B**) The presence of the FOT4 gene in spore clones was analyzed by PCR. A 1 kb DNA Marker was used for size comparison (Nippon Genetics, Düren, Germany). (**C**) Mating-type halo assays were performed with MATa (**left** image) and MATα tester strains (**right** image), as described in Materials and Methods. Control strains indicated as MATa and MATα show a halo as a zone of growth inhibition in a positive interaction; in the left image there is a halo around the MATα colony, and in the right image there is a halo around the MATa colony. However, all spore clones were non-maters as no halos were observed around spore clone colonies. A selection of spore clones is shown here (see also Supplementary Material).

Spore clones were propagated in YPD full medium and then aliquots were spotted on growth plates incubated at 10 °C, 30 °C, and 37 °C; YPD plates containing 1 M NaCl were used to assess osmotic stress tolerance (Figure 2A).

Growth was scored as very good, medium, reduced, or very low to none (Table 2 and Table S1). Mostly, growth at 10 °C was poor (62 strains) to very poor (40 strains), except for one spore clone (GYBC 1459). Spore clones generally grew better at higher temperatures, with 20 strains growing very well even at 37 °C, i.e., better than the CEG parental strain. Growth on salt plates was heterogeneous, revealing nine strains that grew well under this condition. (Full datasets are shown in Figure S1 and Table S1).

#### 3.1.2. Screening of Spore Clones via PCR

It was previously shown that Fungal oligopeptide Transporter (*FOT*) genes contribute to nitrogen utilization of wine yeasts in grape must [33]. Therefore, we analyzed the array of spore clones for the presence of the *FOT4* allele found in CEG. The *FOT4* gene is located on chromosome XVI, which is exclusively contributed by the *S. cerevisiae* parent; however, it is present on only one of the four CHRXVI copies. Correspondingly, *FOT4* was shown to be present in app. one quarter of the spore clones (Figure 2B, Table 2 and Table S1).

Based on these assays, a short list of 21 strains was selected for further characterization. One half of the strains showed superior growth at 37 °C and contained the *FOT4* gene, while the other half represented strains with better growth at lower temperatures often lacking *FOT4* (Table 2). These strains were analyzed for their ability to mate via halo assays against pheromone-sensitive strains. In these assays, all of the selected strains were unable to induce a halo in the haploid tester strains (Figure 2C).

This suggested that these strains are heterozygous at their mating type loci, with both *MATa* and *MATα* alleles present. This was confirmed by PCR (Table 2). Since all spore clones presented themselves as *MATa/α* strains, we quantified their ability to sporulate. However, sporulation frequency of these strains was rather low, except for GYBC 1461 (Table 2).

### 3.2. Fermentation Assays

**Figure 3:**
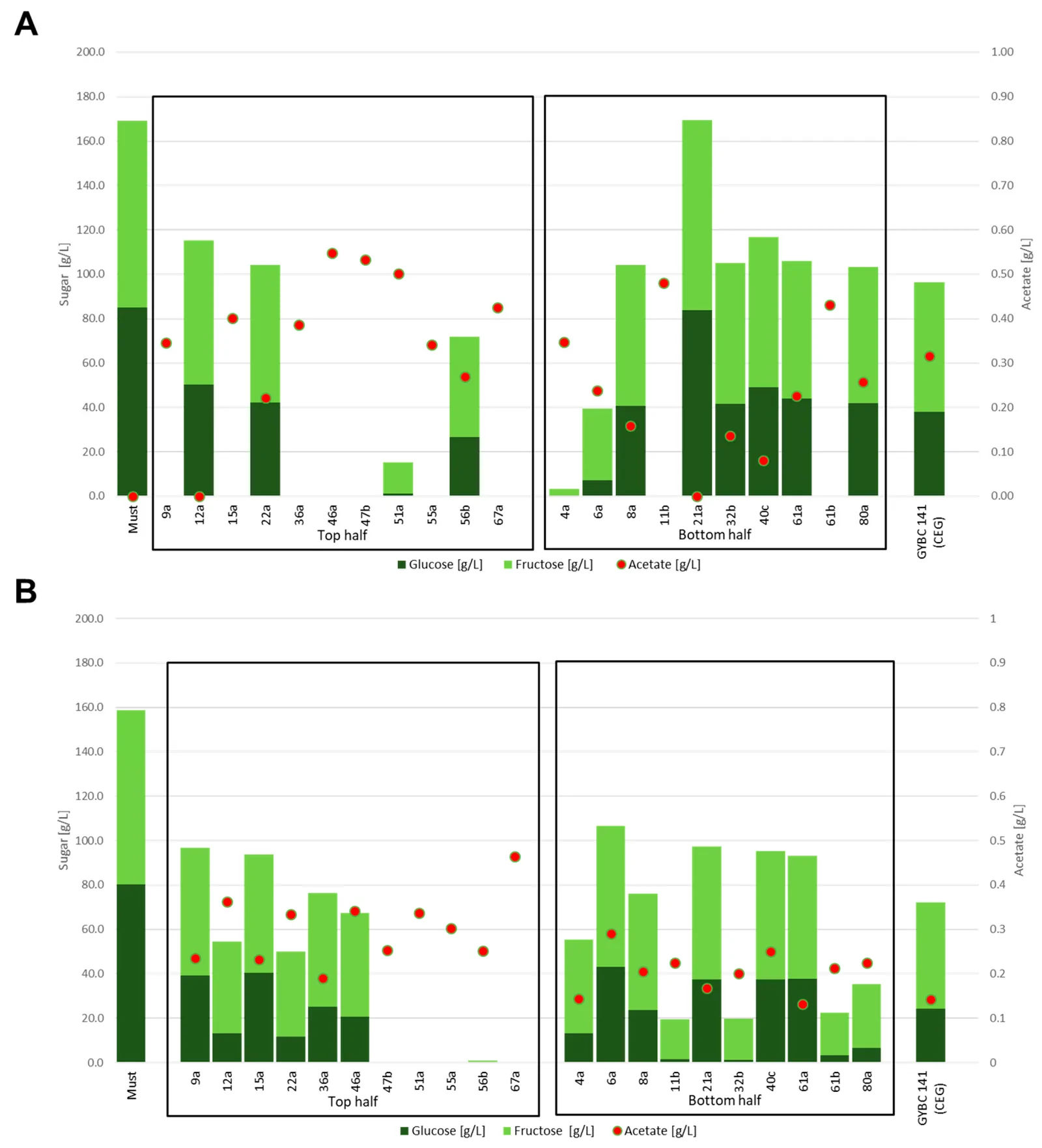
**Single fermentations with selected CEG spore clones.** Fermentations of the selected spore clones listed in Table 2 were run at (**A**) 25 °C and (**B**) 10 °C. Riesling grape must (Must) with low nitrogen content was used. Residual glucose, fructose (bars), and acetate (red dots) levels were determined at the end of fermentation by HPLC. The parental CEG strain was used for comparison.

For an initial characterization of the fermentation capability of the selected strains, grape must fermentations were run at 25 °C for 12 days and at 10 °C for 21 days (Figure 3). We used a 74°Oe Riesling grape must (pH 3.0) from our HGU winery with a decidedly low nitrogen content of 37.1 mg/L ammonium and 64.4 mg/L free amino acids (FAN).

Several of the strains showed sluggish fermentations that stopped with high remaining glucose and fructose levels, while other strains preferentially utilized glucose and retained high fructose concentrations. The parental CEG strain was unable to ferment to dryness and was even more inhibited at 25 °C than at 10 °C (Figure 3).

**Figure 4:**
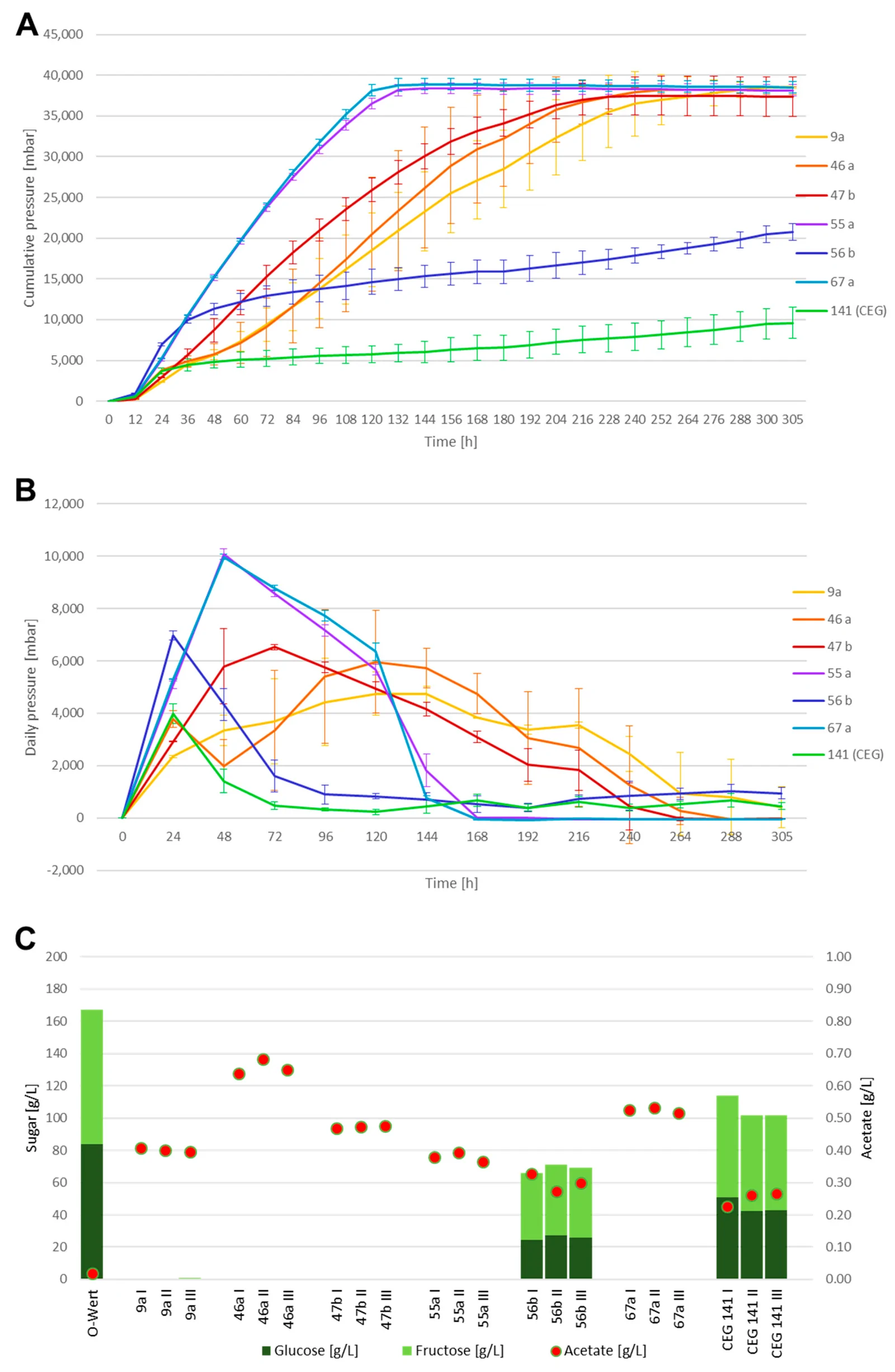
**Triplicate fermentations with improved CEG spore clones.** (**A**) Continuous ANKOM-recorded CO_2_ pressure formation during fermentation. Fermentations of the indicated spore clones and the CEG parental strain were run in triplicate at 25 °C. (**B**). Daily pressure [mbar] monitored during the fermentation of the spore clones and the CEG parental strain. (**C**). Residual glucose, fructose (bars), and acetate (red dots) levels were determined by HPLC at the end of fermentation.

Fermentations were monitored continuously via CO_2_ pressure build-up (Figure S2). At the end of fermentation, amounts of glucose/fructose organic acids and ethanol were measured by HPLC (Tables S2 and S3). Nine of the strains finished the 25 °C fermentations and fermented the grape must to dryness, but only five strains finished their 10 °C fermentations, i.e., the amount of residual sugars was ≤1 g/L at the end of fermentation. Interestingly, three strains were able to finish fermentations at both temperatures, GYBC 1465 (47b), GYBC 1467 (55a) and GYBC 1471 (67a) showing clear improvements over the CEG parental strain. Acetate levels were lower in the 10 °C fermentations, but remained below 0.6 g/L in the 25 °C fermentations (Figure 3). With these data we selected six strains for a second round of fermentations (see Materials and Methods, Figure 4).

These strains included the three strains that completed both the 10° and 25° fermentations. Additionally, GYBC 1455 (9a) and GYBC 1464 (46a) completed the 25 °C fermentations but not the 10 °C fermentations, whereas GYBC 1468 (56b) completed only the 10 °C fermentations. These strains and CEG were fermented in triplicates at 25 °C using the same Riesling must as before. As before, fermentations were monitored with ANKOM-based measurements of CO_2_ pressure and HPLC analytics were performed at the end of fermentation (Figure 4, Table S4).

CEG and GYBC 1468 (56b) exhibited very sluggish fermentations. In contrast, GYBC 1455 (9a), GYBC 1464 (46a), and GYBC 1465 (47b) showed slower fermentation but ultimately completed fermentation within 12 days, similar to the faster-fermenting strains. GYBC 1467 (55a) and GYBC 1471 (67a) were the fastest fermenting strains. Even over the four-day period (t = 48 h to t = 144 h), these two spore clones showed the highest values for daily pressure release (Figure 4B). After 24 h, the strain GYBC 1486 (56b) had achieved the highest daily pressure release (6966.79 mbar). This then decreased sharply as fermentation progressed, remaining nearly constant after 96 h. Of all the strains tested during fermentation, CEG produced the lowest maximum daily pressure release after 24 h (3980.18 mbar). This value then decreased sharply before leveling off after 72 h. Acetate levels were low in GYBC 1455 (9a) and GYBC 1467 (55a) and above 0.6 g/L in GYBC 1464 (46a). These data clearly demonstrate that meiotic selection resulted in spore clones with improved fermentation characteristics compared with CEG. Figure S3 illustrates the duration of the lag phase by showing a graph of the cumulative pressure measured during the fermentation of CEG and the spore clones over the first eight hours.

### 3.3. Fermentation Kinetics

The maximum amount of CO_2_ produced after 305 h of fermentation was calculated, as was the time at which 50% of the maximum CO_2_ level was reached (Table 3). All of the spore clones produced higher levels of CO_2_ compared with the CEG parental strain. Within the first 60 h of fermentation, the CO_2_ yield of CEG and three of the spore clones (GYBC 1467, GYBC 1468, and GYBC 1471) reached 50% of its maximum yield. In contrast, GYBC 1455 and GYBC 1464 exceeded this threshold after over 100 h of fermentation.

The maximum relative growth rate (µ_rel.max_) was determined in order to analyze the increase in CO_2_ production in relation to the level of CO_2_ already produced (Table 3). The maximum relative growth rate of all strains was detected in the beginning of the fermentation (t = Δ1/Δ2). CEG and three of the spore clones (GYBC 1467, GYBC 1468, and GYBC 1471) showed high levels of maximum growth rate (>1.00 h^−1^), while three spore clones revealed lower maximum relative growth rates (<1.00 h^−1^). The peak CO_2_ production rate of the latter three strains was reached after 190 h, whereas the peak CO_2_ production rates of all the other tested yeast strains were detected within the first 40 h of fermentation (Table S5).

### 3.4. Aroma Analyses

**Figure 5:**
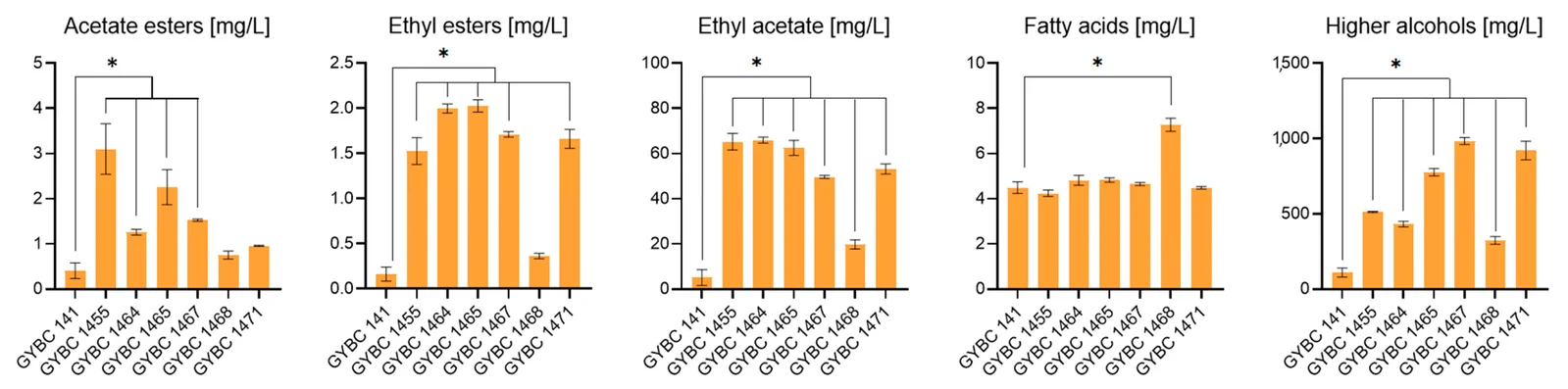
**Fermentation aromas generated by CEG and its spore clones.** Different aroma classes are summarized and bar heights indicate the average sum of all analytes per class; error bars indicate class mean ± standard error of the mean. Asterisks indicate that CEG volatile aroma amounts were significantly different from the indicated strains as determined by a classical ANOVA, including a homogeneity test with Welch’s correction and a post hoc test using the Games–Howell test with * p  <  0.05.

At the end of fermentation, we analyzed different aroma compounds via GC-MS (Figure 5). As CEG and GYBC 1468 (56b) did not complete their fermentation it was not surprising that also the aroma production was very low in these strains. The other strains finished their fermentations and GYBC 1467 (55a) stood out with low ethyl acetate levels <60 mg/L and increased levels of higher alcohols, mainly isoamyl alcohol. This strain, GYBC 1464 (46a), GYBC 1465 (47b), and GYBC 1471 (67a) produced favorable amounts of ethyl esters of up to 2 mg/L with higher amounts of ethyl octanoate and similar amounts of ethyl butyrate and ethyl hexanoate (Figure 5 and Figure S4). GYBC 1455 (9a) was the most prominent acetate ester producer, particularly of isoamyl acetate and 2-phenylethyl acetate (Figure S4).

**Figure 6:**
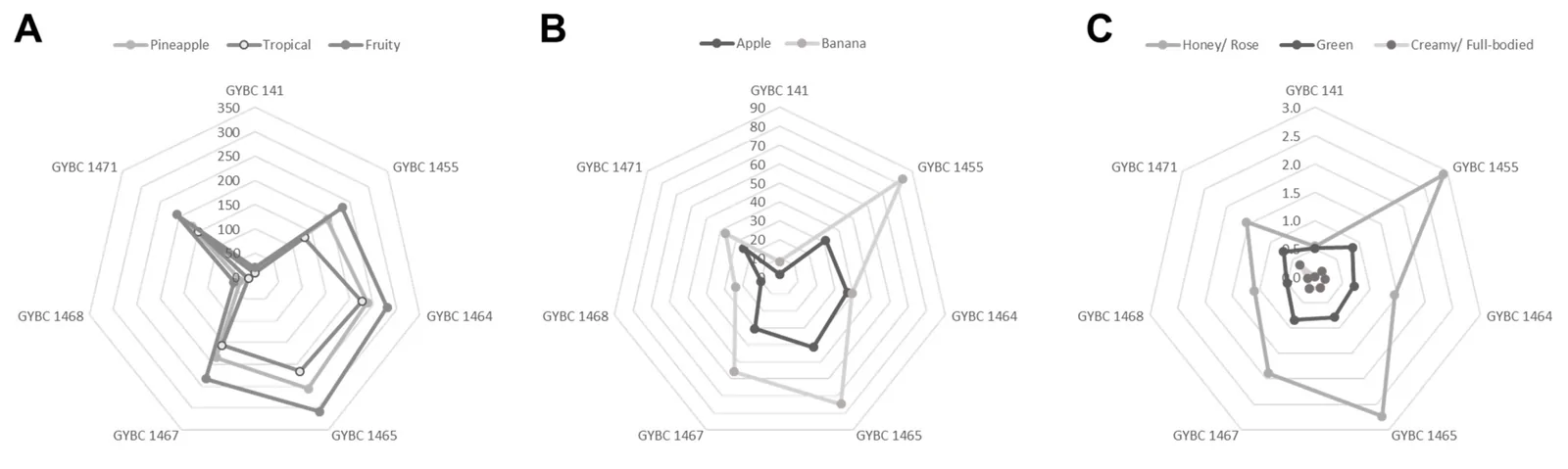
**Aroma profiles of wines produced by CEG and its spore clones.** (**A**–**C**) OAV data are based on the triplicate fermentations in Riesling must at 25 °C (see Tables S8 and S9 for key aroma compounds included with the descriptors). Please note that OAVs do not directly indicate sensory quality because they do not consider matrix effects or interactions between compounds.

Odor activity values (OAVs) were calculated by dividing the concentration of each aroma compound by its respective detection threshold [34,35,36,37,38,39,40]. This allows the graphical processing of aroma data according to sensory aroma descriptors. The descriptors used were pineapple, tropical, fruity, apple, banana, honey/rose, green, and creamy for which distinct volatile aroma compounds were combined (Figure 6, Tables S6–S9) [34,35,36,37,38,39,40].

GYBC 1464 produced high levels of pineapple and tropical–fruity aromas, but generated lower levels of banana and honey/rose aromas than GYBC 1455, GYBC 1465 and GYBC 1467. GYBC 1455 peaked in these two categories. However, GYBC 1464 showed a longer lag phase at the beginning of fermentation and required a considerably longer overall fermentation time than the faster strains.

Overall, GYBC 1455, GYBC 1464, GYBC 1465, and GYBC 1467 delivered acetate and ethyl esters in these 25 °C fermentations in amounts similar to previous fermentations with CEG at 18 °C [14]. Combined with fermentation speed and low levels of acetate formation, GYBC 1467 (55a) appeared to be the favorite strain in this selection.

### 3.5. Qualitative Indication of β-Glucosidase Activity

**Figure 7:**
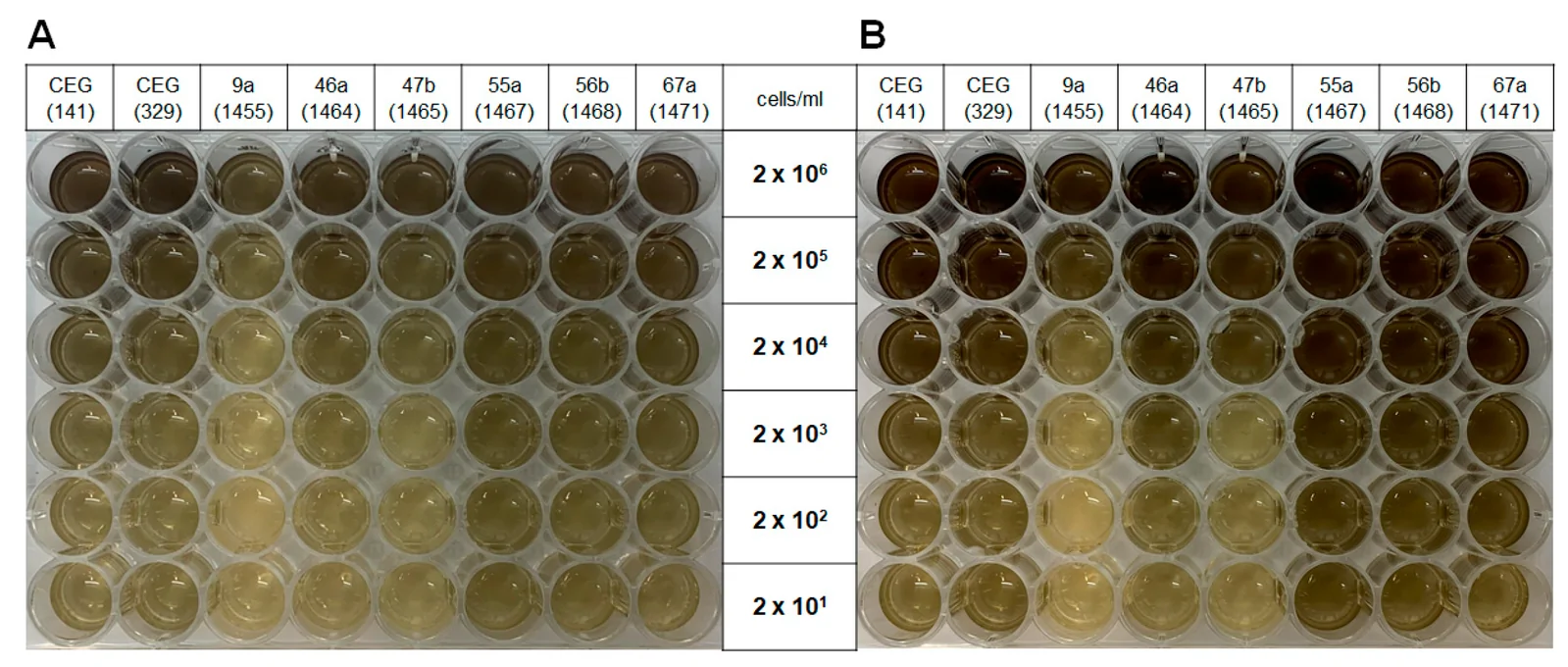
**Qualitative assay for β-glucosidase activity.** Ten-fold serial dilutions of the indicated strains were prepared with esculin medium and incubated at 25 °C. Images of the same plate were acquired after (**A**) two days and (**B**) three days. Darkness of wells correlates with β-glucosidase activity.

A major fraction of the flavor compounds is sequestered as odorless non-volatile glycosides [41,42]. Therefore, β-glucosidase activity was assessed in a qualitative assay using esculin as a substrate, which after hydrolysis yields esculetin and forms a black complex with ferric ions [43]. Ten-fold serial dilutions revealed different β-glucosidase activities in the spore clones tested (Figure 7 and Figure S5).

CEG exhibited medium activity levels, while GYBC 1455 (9a) and GYBC 1465 (47b) showed lower β-glucosidase activities. GYBC 1467 (55a) and GYBC 1468 (56b) showed stronger activity in these assays.

### 3.6. Genome Analyses of Spore Clones

**Figure 8:**
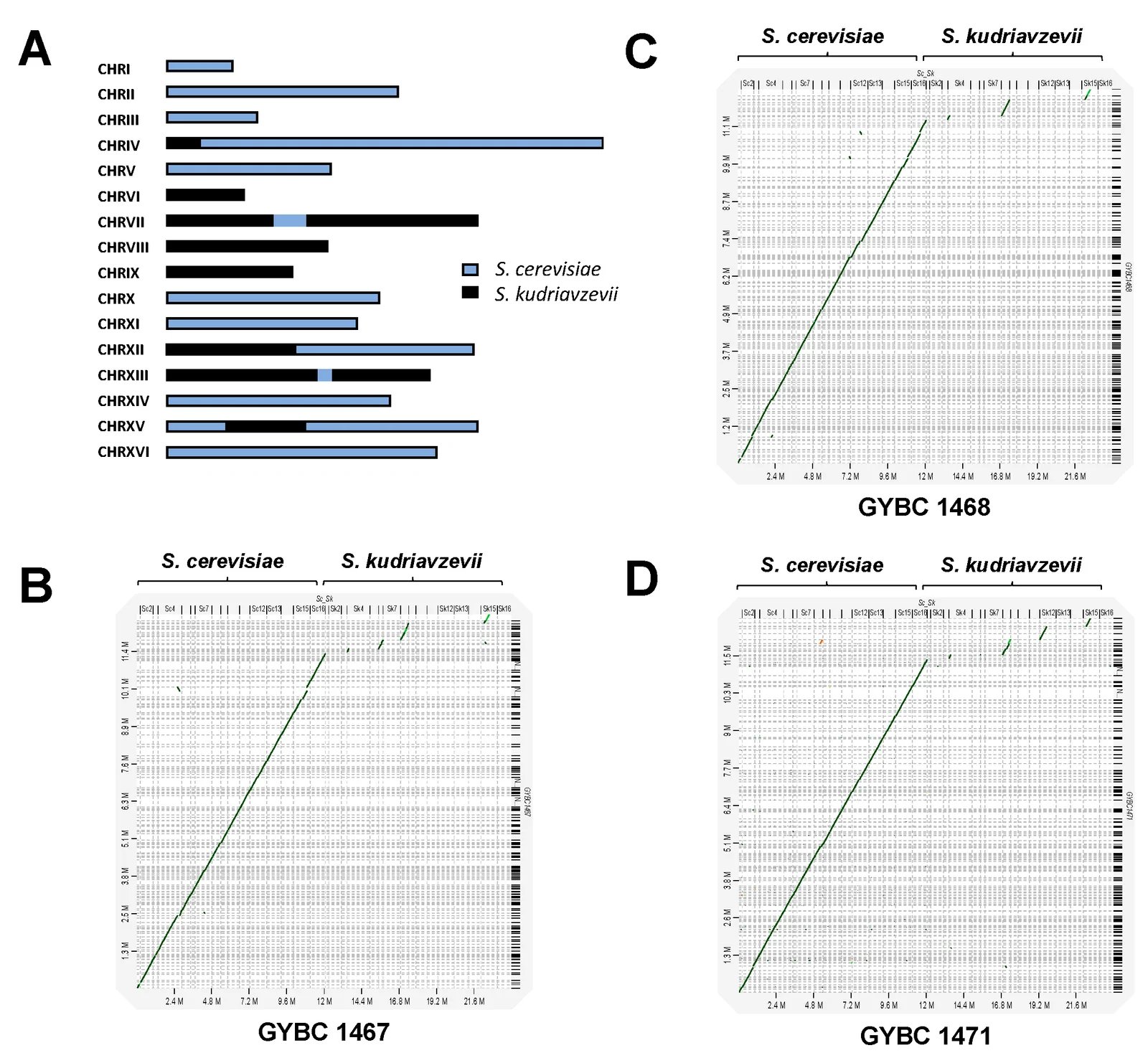
**Genome analyses of CEG spore clones.** (**A**) The original *S*. *kudriavzevii* genome content of CEG mapped onto the 16 *Saccharomyces* chromosomes as determined previously [14]. Note that the parental CEG strain contains an additional three genome equivalents of *S*. *cerevisiae* DNA. (**B**–**D**) Dot plot genome comparisons of the spore clone genomes against *S*. *cerevisiae* (S288C, https://www.yeastgenome.org/) and *S*. *kudriavzevii* (IFO1802, GCA_000167075.2) genomes were generated by comparing contigs using D-GENIES with default settings [45]. Matching contigs appear as a line indicating full *S*. *cerevisiae* genomes in all spore clones with smaller *S*. *kudriavzevii* content (Table 4).

One of our selection criteria for CEG-derived spore clones was improved fermentation at higher temperatures. Since *S. cerevisiae* contributes the dominant part of the CEG genome, we expected that meiotic divisions and viable spore clone formation resulted in a loss of *S. kudriavzevii* chromosomes as has been observed, for example, in *S. cerevisiae/S. uvarum* hybrids [44]. To test this, we used Illumina NextSeq 2000 whole genome sequencing and compared the genomic composition of three selected spore clones, GYBC 1467 (55a), GYBC 1468 (56b), and GYBC 1471 (67a), with the CEG genome assembly (Figure 8).

Sequencing reads were assembled into contigs and scaffolds and these were blasted against the *S. cerevisiae* and *S. kudriavzevii* genomes (https://blast.ncbi.nlm.nih.gov/Blast.cgi, accessed on 6 February 2026). This approach was sufficient to determine the absence or presence of the *S. kudriavzevii* DNA contigs in the spore clones in comparison to those found in CEG due to the low sequence identity between *S. cerevisiae* and *S. kudriavzevii* (~85%). Genes were annotated for each contig and the contigs aligned to chromosomes. These data were compared with the original CEG genome analysis [14]. While all spore clones contained contig DNA of all *S. cerevisiae* chromosomes, the contribution of *S. kudriavzevii* was drastically reduced in these strains. Based on the mapping data, loss of the *S. kudriavzevii* chromosomes VII, IX, and XIII was detected in all strains. *S. kudriavzevii CHRVI* was lost in GYBC 1468 (56b) and GYBC 1471 (67a) but retained in GYBC 1467 (55a). The *S. cerevisiae/S. kudriavzevii* hybrid chromosome *CHRXII* was lost in GYBC 1467 (55a) and GYBC 1468 (56b) but retained in GYBC 1471 (67a) (Table 3). In our previous analysis, we estimated the *S. kudriavzevii* contribution to the CEG genome to be ~3850 kb [14]. This contribution was now reduced to ~1240 kb in GYBC 1467 (55a), ~970 kb in GYBC 1468 (56b), and ~1420 kb in GYBC 1471 (67a).

**Table 4:** *S. kudriavzevii* contribution to the genomes of CEG spore clones.

Chromosome	Interval Description	GYBC 1467 *	GYBC 1468	GYBC 1471
IV	140 kb between YDL241w and YDL179w	Present	Present	Present
VI	Complete chromosome	Present	Lost	Lost
VII	1090 kb, YGL253w to YGR295c; ScDNA gap 112.8 kb between YGL098w and YGL033w	Lost	Lost	Lost
VIII	Complete chromosome	Present	Present	Present
IX	Complete chromosome c	Lost	Lost	Lost
XII	Left end up to rDNA repeats; 448 kb, YLL067c to YLR154c	Lost	Lost	Present
XIII	924 kb, YML131w to YMR319c; ScDNA gap 44.8 kb between YMR140w and YMR166c	Lost	Lost	Lost
XV	270 kb between YOL056w and YOR091W	Present	Present	Present

*: present: sequence retained as in CEG background, lost: sequence absent in spore clone.

## 4. Discussion

Changing requirements for fermentation yeasts include increased resilience to altered grape must composition driven by climate change—particularly elevated sugar concentrations (>240 g/L) and reduced acidity (<6 g/L tartaric acid)—and effects on the aroma precursor composition alongside persistent consumer demands for enhanced aroma complexity [46,47]. Yeast breeding is the major non-GMO tool to fill this need without genetic engineering [14]. By combining the genetic potential of different *S. cerevisiae* starter strains, yeast breeding generates specialized cultures tailored for industrial fermentation [48,49]. Breeding can be used to generate stable hybrids of the same or different *Saccharomyces* species, select progeny that outperform their parents, e.g., by improved growth at higher temperature, higher osmolarity, or higher alcohol levels [50]. Alternatively, non-conventional yeasts, i.e., non-*Saccharomyces* yeasts can be employed in co-fermentations. A growing number of these yeasts are available as starter cultures [18,19]. However, co-fermentations are less predictable and less consistent than single strain pure culture fermentations.

Yeasts of the genus *Saccharomyces* have retained the ability to form interspecies hybrids, albeit with a low frequency indicating a considerable prezygotic barrier in sexual crosses [51,52]. Additionally, postzygotic barriers exist in hybrids that lead to low sporulation frequencies and low spore viability [53]. The CEG wine yeast was characterized as a hybrid between *S. cerevisiae* and *S. kudriavzevii* with a dominant *S. cerevisiae* genome [14]. Thus, it already represents an evolved hybrid that has reduced the *S. kudriavzevii* parental genome, e.g., via GARMe (genome auto-reduction by meiosis, [54]). To distinguish *bona fide* hybrids from evolved hybrids, the term chimera has been introduced [53]. Chimeras similar to CEG have been described from different fermentation environments [55,56,57]. The degree of *S. kudriavzevii* genome exclusion in these strains varies, but it has been suggested that remnants of the *S. kudriavzevii* genome may be required for adaptation to low fermentation temperatures, glycerol production, or complex aroma formation [58]. Chimera with *S. kudriavzevii* mitochondrial DNA appeared to hold a larger part of the *S. kudriavzevii* nuclear genome and, thus, chimera with the *S. cerevisiae* mitochondrial genome was able to lose larger parts of *S. kudriavzevii* DNA, such as the tetraploid *Assmanshausen* red wine yeast [59].

The CEG strain is able to sporulate and yield viable spores, although at a reduced rate. The frequency of viable spores was <20%, indicating difficulties in meiosis. The viable spores generated novel and diverse spore clones, i.e., new chimeras that differ from their parental strain and from each other due to meiotic segregation of chromosomes. Genome sequencing of three of the spore clones indicated further and differential loss of *S. kudriavzevii* DNA in these strains. The original CEG strain harbors ~3.85 Mb of *S. kudriavzevii* DNA while only 0.97–1.42 Mb of *S. kudriavzevii* DNA was retained in the genomes of three of the sequenced spore clones. The CEG spore clones were infertile and exhibited a very low sporulation rate (except GYBC 1461), suggesting that these *MATa/MATα* strains are at least diploids displaying postzygotic barriers.

From a pool of >120 spore clones, we selected strains with improved fermentation characteristics compared with the CEG strain, particularly improved fermentation of nitrogen-poor musts at higher temperature. Conceptually, this may reverse the original benefit of an *S. cerevisiae/S. kudriavzevii* hybrid with better performance at lower temperatures [17]. Genes implicated in low-temperature tolerance are involved in glycerol biosynthesis (*PGI1*, *GPD1*, and *TIP1*), sterol synthesis (*ARE1*), *GUT2* (a mitochondrial glycerol-3-phosphate dehydrogenase), and *ADH3* (a mitochondrial alcohol dehydrogenase) [17,60,61]. In CEG, *TIP1* (YBR067c), *PGI1* (YBR196c), *GPD1* (YDL022w), and *ARE1* (YCR048W) are encoded only by the *S. cerevisiae* orthologs. *GUT2* (YIL155c) and *ADH3* (YMR083w) are present as *S. cerevisiae* and *S. kudriavzevii* copies in CEG. In three spore clones selected for superior fermentation at elevated temperature (GYBC 1467, GYBC 1468, and GYBC 1471), genomics indicated loss of *SkCHRIX* and *SkCHRXIII* and thus loss of the *S. kudriavzevii* alleles of *GUT2* and *ADH3.* However, the selected spore clones performed well both at 10 °C and 25 °C suggesting that additional mechanisms are relevant and loss of *S. kudriavzevii* DNA alone is not sufficient to explain the advantages of the spore clones. This observation is hypothesis-generating and cannot establish a causal relationship because only three spore clones were sequenced and no reciprocal genotype–phenotype validation was performed.

In CEG, an allele of a fungal oligopeptide transporter gene, *FOT4,* is present in a single copy on CHRXVI. However, CEG showed a very poor fermentation in nitrogen-limited Riesling must. The selected spore clones, however, very efficiently fermented this low-nitrogen Riesling must. To what extent *FOT4* may have contributed to this is unclear and requires further investigation into the *FOT4* copy number in the different strains, and the actual characterization of peptide utilization via Fot4p. The importance of *FOT* genes in nitrogen acquisition has already been demonstrated, e.g., in the wine yeast EC1118 [31,62,63].

After reaching its maximum daily pressure level within 24 h, CEG enters a stuck fermentation after 72 h, similar to GYBC 1468. This involves a peak after 24 h, followed by the onset of stagnant fermentation after 96 h. Both yeast strains produced half of their total CO_2_ output within the first 50 h. They reached their maximum CO_2_ production rate after 16–17 h and achieved the highest relative growth rate of all the strains tested at the start of fermentation. This demonstrates that the microorganisms are functioning efficiently, initiating fermentation swiftly, and that the rate of CO_2_ production increases most rapidly at the beginning of the process (when CO_2_ levels are low).

By contrast, the three spore clones GYBC 1455, GYBC 1464, and GYBC 1465 demonstrate consistently higher daily pressure release over several days of fermentation (48–240 h). These yeast strains initiate fermentation more slowly, only reaching peak activity in the second half of the process. Evidence of this can be seen in the lowest relative growth rates detected among all the yeast strains tested, as well as in the highest CO_2_ production rate 190 h after fermentation began and in the very late stage at which 50% of the total CO_2_ had been produced.

In terms of fermentation kinetics, two spore clones stand out in particular: GYBC 1467 and GYBC 1471 exhibit the highest daily pressure release values, ranging from 5.7 to 10.1 bar over a period of four days, indicating very high microbial activity. This observation is supported by the high relative growth rate at the start of fermentation, the maximum CO_2_ production rate around the 20 h mark, and the fact that 50% of the total CO_2_ had already been produced after 60 h of fermentation. In contrast to CEG, both strains completed fermentation successfully and outperformed CEG in terms of kinetics. This resulted from a lag phase that was 1–2 h shorter and produced more CO_2_.

Improved strains utilized the available sugars in must and generated the expected amount of ethanol (~10% vol/vol). The CEG spore clones should also be able to generate interesting and preferable aroma in white wine. Compared with our previous characterization of CEG in fermentations at 18 °C [14], the new spore clones produced higher amounts of higher alcohols, acetate esters, and ethyl acetate, but lower amounts of fatty acids and ethyl esters during fermentations at 25 °C. This is in line with previous reports that higher temperature fermentations promote these changes in aroma composition [64,65].

When analyzing volatile aroma compounds, there are several factors to bear in mind. All components have different odor thresholds, which can vary depending on the matrix. Due to their lower odor threshold, esters can have a greater impact on the aroma profile of wines than higher alcohols, although their presence in smaller quantities and their concentration decreases through chemical hydrolysis during the aging process.

Fermentations conducted at higher temperatures, as in this study, tend to produce increased levels of volatile aroma compounds [64,66]. The quantity of an aroma component produced can determine whether it results in a pleasant or unpleasant aroma. For example, ethyl acetate can contribute to a pleasant, fruity aroma in concentrations of less than 100 mg/L [67]. However, if the concentration exceeds this level, the aroma becomes unpleasant and solvent-like, spoiling the wine. All fermentations in this study showed ethyl acetate levels below 100 mg/L, a range that tends to have a positive effect on overall aroma. Furthermore, the total ester content (the sum of all ethyl and acetate esters) does not exceed 200 mg/L in any fermentation process involving any of the tested strains, indicating that esters can impart a fruity or floral aroma [68].

Total higher alcohol concentrations below 300 mg/L can have a positive contribution to the overall complexity of the final wine, whereas concentrations exceeding 400 mg/L can have a negative impact [67,68]. All the tested strains contain higher concentrations of total higher alcohols, except for CEG and GYBC 1468, which did not finish fermentation. Therefore, future studies that include sensory validation through descriptive analysis or consumer testing could clarify the potential impact on the overall aroma and confirm the perception of flavor profiles.

Besides higher alcohols and esters, the acetic acid content plays an important role in the aroma profile and the overall acceptance of wines. Except for CEG, all strains tested exceeded the odor threshold of acetic acid (280 mg/L) [69]. GYBC 1467 showed the lowest acetic acid levels of all the strains that finished fermentation. Again, a sensory evaluation could be performed in a follow-up study to determine the acceptance level of acetic acid.

Since only laboratory-scale fermentations were performed, upscaling these fermentations and conducting more detailed analyses, including sensory analyses by taste panels, are required for a direct comparison of fermentations between CEG and its spore clones. Additional qualitative assays for β-glucosidase activity using esculin as a substrate suggested varying activity levels in the spore clones compared with CEG, with increased activity observed in GYBC 1467 and GYBC 1468 and decreased activity in GYBC 1455 and GYBC 1465. A large number of non-volatile, glycosylated aroma precursors are terpenoids [70]. CEG spore clones with higher β-glucosidase activity may therefore release larger amounts of these aroma compounds than CEG, an effect that remains to be quantified, including in upscaling experiments. However, the esculin assay is qualitative and was not performed in a wine fermentation matrix. Additional experiments, such as the quantification of β-glucosidase activity in wine fermentations, are required to determine the relationship between β-glucosidase activity and potential aroma improvement.

Two strains that surpassed CEG and generated very pleasant tropical, fruity aromas were GYBC 1467 and GYBC 1471. Both strains are fast fermenting, robust strains that show considerable improvement over the parental CEG strain.

## 5. Conclusions

Yeast breeding offers a convenient, non-GMO tool for strain improvement of wine yeasts. Sexual crosses require strains that either have the ability to mate or generate viable spores that can be clonally amplified. Yeast breeding can utilize pure strains or chimeras that circumvented postzygotic barriers, e.g., by tetraploidy or genome autoreduction. Molecular yeast breeding uses genomic information and allows the generation of hybrids or spore clones that combine specific alleles, shown here by the selection of spore clones harboring a fungal oligopeptide transporter gene (*FOT4*). While breeding efforts benefit from stable, haploid, and heterothallic strains, we have shown in that complex strains, such as aneuploid/tetraploid chimeric strains, can also be improved and adapted to challenging fermentation conditions.

## Figures and Tables

**Table 1:** Isolation of CEG spore clones.

Plate	Number of Tetrads	Spores Isolated	Colonies Formed per Tetrad					Number of Colonies
			0	1	2	3	4	
A	20	80	12	7	1	0	0	9
B	20	80	12	7	1	0	0	9
C	10	40	8	1	1	0	0	3
E	10	40	7	2	1	0	0	4
F	10	40	6	3	1	0	0	5
G	10	40	5	3	1	1	0	8
H	10	40	6	1	3	0	0	7
I	10	40	4	3	3	0	0	9
J	18	72	3	10	4	1	0	21
K	18	72	10	3	5	0	0	13
L	10	40	5	3	2	0	0	7
M	16	64	3	6	7	0	0	20
N	16	64	8	7	1	0	0	9
Sum	178	712	89	56	31	2	0	124
%			50	31.46	17.42	1.12	0	

**Table 2:** Selection of CEG spore clones.

Set	Strain Number	37 °C #	10 °C	30 °C	30 °C + 1 M NaCl	Sporulation (%)	*FOT* Gene +	*MAT* Halo Assay	Fermentation	
									25 °C *	10 °C
A	GYBC 1468, 56b	3	2	3	3	1.0	YES	a/α		fin.
A	GYBC 1460, 22a	2	2	3	2	1.0	YES	a/α		
A	GYBC 1458, 15a	1	2	2	3	3.5	YES	a/α	fin.	
A	GYBC 1466, 51a	2	2	2	2	0.5	YES	a/α		fin.
A	GYBC 1464, 46a	3	1	3	3	5.5	YES	a/α	fin.	
A	GYBC 1467, 55a	3	1	3	2	0.5	YES	a/α	fin.	fin.
A	GYBC 1457, 12a	3	1	3	1	2.0	YES	a/α		
A	GYBC 1455, 9a	3	1	3	0	3.0	YES	a/α	fin.	
A	GYBC 1471, 67a	3	1	2	2	1.0	YES	a/α	fin.	fin.
A	GYBC 1465, 47b	3	1	2	1	2.5	YES	a/α	fin.	fin.
A	GYBC 1462, 36a	3	0	2	2	0.5	YES	a/α	fin.	
B	GYBC 1463, 40c	2	2	3	3	0.0	n.d.	a/α		
B	GYBC 1469, 61a	2	2	3	3	0.0	n.d.	a/α		
B	GYBC 1470, 61b	2	2	3	2	0.5	n.d.	a/α	fin.	
B	GYBC 1461, 32b	2	2	3	2	33.0	n.d.	a/α		
B	GYBC 1472, 80a	1	2	2	1	0.0	n.d.	a/α		
B	GYBC 1459, 21a	0	3	3	2	0.5	n.d.	a/α		
B	GYBC 1454, 8a	0	1	3	1	0.0	n.d.	a/α		
B	GYBC 1452, 4a	0	1	3	0	1.0	YES	a/α		
B	GYBC 1453, 6a	0	1	2	1	0.5	n.d.	a/α		
B	GYBC 1456, 11b	0	1	2	0	0.0	YES	a/α	fin.	
	CEG	2	1	3	1	5.5	YES	a/α		

#: Growth of colonies was scored under different conditions as either very good (3), medium (2), reduced (1), or very low/no growth (0). +: n.d.: not detected. *: fin.: finished fermentations contain ≤1 g/L residual glucose or fructose.

**Table 3:** Fermentation kinetics of the spore clones and CEG parental strain.

GYBC	n(CO_2_)_max_ [Mol]	t_50%_ CO_2_ Production [h]	µ_rel.max_ [h^−1^]
141	6.43 × 10^−4^ ± 1.16 × 10^−4^	48.51 ± 18.66	1.75 ± 0.25
1455	2.34 × 10^−3^ ± 1.15 × 10^−5^ *	124.89 ± 23.25	0.57 ± 0.08 *
1464	2.31 × 10^−3^ ± 2.65 × 10^−5^ *	117.31 ± 29.08	0.88 ± 0.11
1465	2.26 × 10^−3^ ± 1.47 × 10^−4^ *	85.91 ± 8.35	0.90 ± 0.17
1467	2.31 × 10^−3^ ± 4.58 × 10^−5^ *	58.42 ± 1.70	1.26 ± 0.31
1468	1.26 × 10^−3^ ± 6.24 × 10^−5^ *	39.87 ± 3.36	1.63 ± 0.20
1471	2.33 × 10^−3^ ± 4.58 × 10^−5^ *	58.65 ± 0.71	1.55 ± 0.18

Results are presented as the mean  ±  SD of three biological replicates. Significant differences in n(CO_2_)_max_ [Mol], t_50%_ of CO_2_ production [h], and maximum relative growth rate (µ_rel.max_; [h^−1^]) between the spore clones and their parental strain (GYBC 141) were determined using a classical ANOVA, including a homogeneity test with Welch’s correction and a post hoc test using the Games–Howell test, * *p*  <  0.05 as compared with the control.

## Data Availability

The data presented in this study are openly available in Genbank at https://www.ncbi.nlm.nih.gov/genbank/ (accessed on 17 March 2026).

## Supplementary Materials

The following supporting information can be downloaded at: https://www.mdpi.com/article/10.3390/microorganisms14081679/s1, Figure S1: Characterization of spore clones; Figure S2: Single fermentations with selected CEG spore clones; Figure S3: Triple fermentations with CEG and selected CEG spore clones of the first eight hours of fermentation; Figure S4: Aroma formation of selected CEG spore clones; Figure S5: Qualitative β-glucosidase assay. Table S1: Growth assays with CEG spore clones; Table S2: HPLC dataset for single fermentations run at 25 °C; Table S3: HPLC dataset for single fermentations run at 10 °C; Table S4: HPLC dataset for triplicate fermentations run at 25 °C; Table S5: Maximum production rate of CO_2_ (m(CO_2_)_max_ [Mol/h]) and the time of detection (t_m(CO2).max_ [h]) of CEG and all spore clones during the fermentation in triplicates; Table S6: Quantification of aroma compounds; Table S7: Odor thresholds of Higher alcohols, acetate esters and ethyl esters, and their possible sensory effects on the aroma profile of wines; Table S8: Aroma formation [OAV]^Δ^ during the fermentations using CEG and its spore clones; Table S9: The calculated odor active value [34,35,36,37,38,39,40] for the respective higher alcohols, acetate esters and ethyl esters.
