# Supplementary material for: Improvement of the Champagne–Epernay–Geisenheim (CEG) Wine Yeast via Meiotic Spore Clone Selection
Source: Microorganisms. 2026 Jul 31;14(8):1679. doi: 10.3390/microorganisms14081679 (PMC13515970; doi:10.3390/microorganisms14081679)
Supplement: Supplementary file 1 [file microorganisms-14-01679-s001.zip › microorganisms-4361363-supplementary.pdf]

Supplementary materials

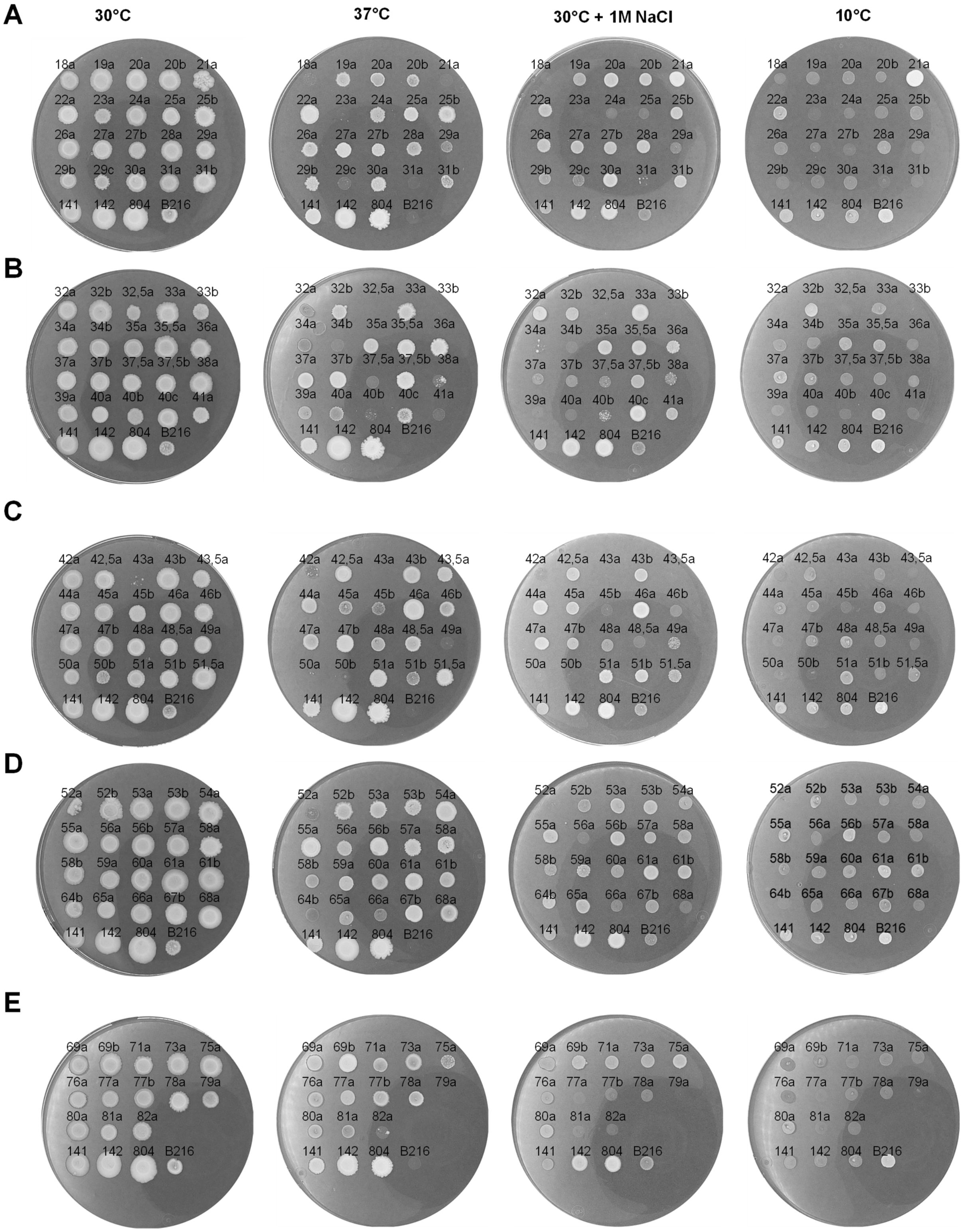

Figure S1. Characterization of spore clones. (A-E) *Spore clones were spotted on YPD plates or YPD-plates containing 1 M NaCl, incubated for six days at the indicated temperatures and then photographed. The bottom row of each plate contains control strains: 141: CEG; 142: Geisenheim wine yeast GHM; 804: EC1118 wine yeast; B216: CBS 8840 Saccharomyces kudriavzevii.*

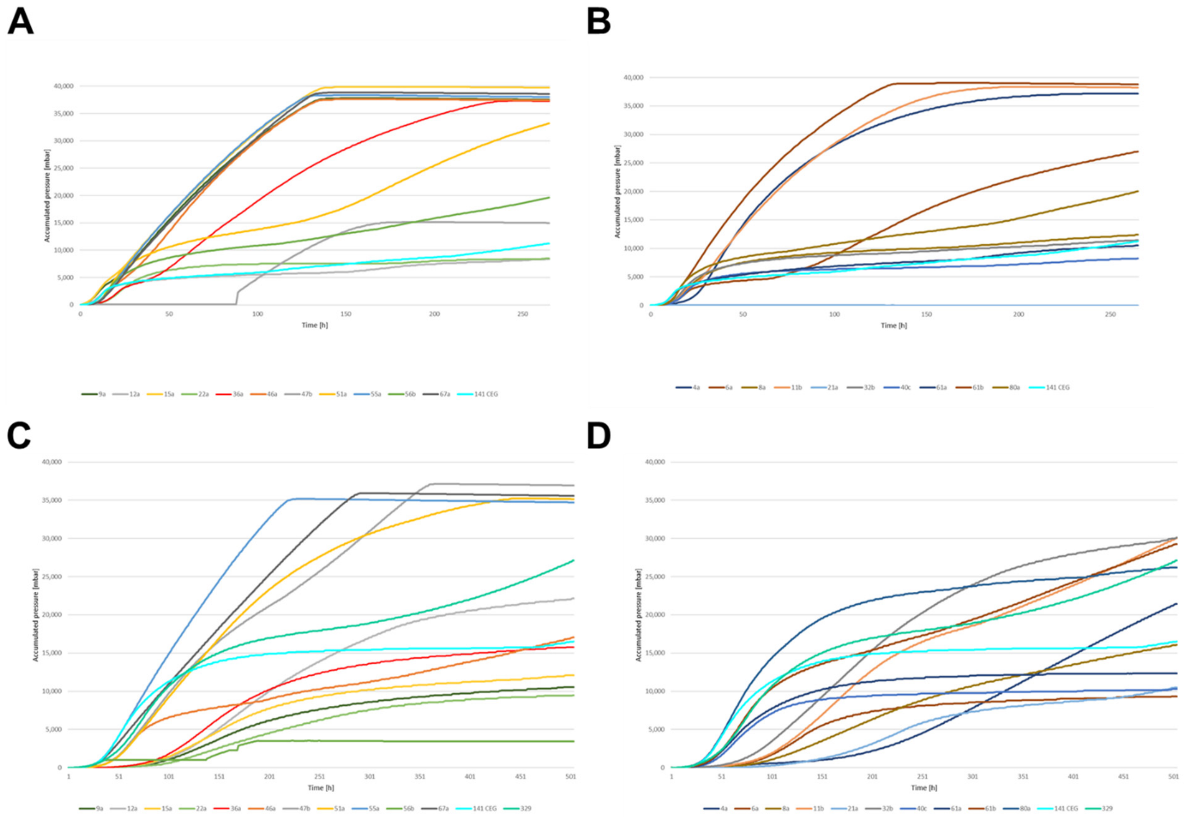

Figure S2. Single fermentations with selected CEG spore clones. *Ankom fermentations were monitored continuously to record* CO_2 *pressure formation. Fermentations of the selected spore clones listed in Table 2 were run at 25°C **(A,** set A; **B,** set B**)** and 10°C **(C,** set A; **D,** set B**)**. The parental CEG (#141) was used for comparison. Pressure recordings for strains 47b in **2A a**nd 56b in **2C** failed, apparently, yet HPLC data indicated that these strains actually finished their fermentations (Tables S2, S3).*

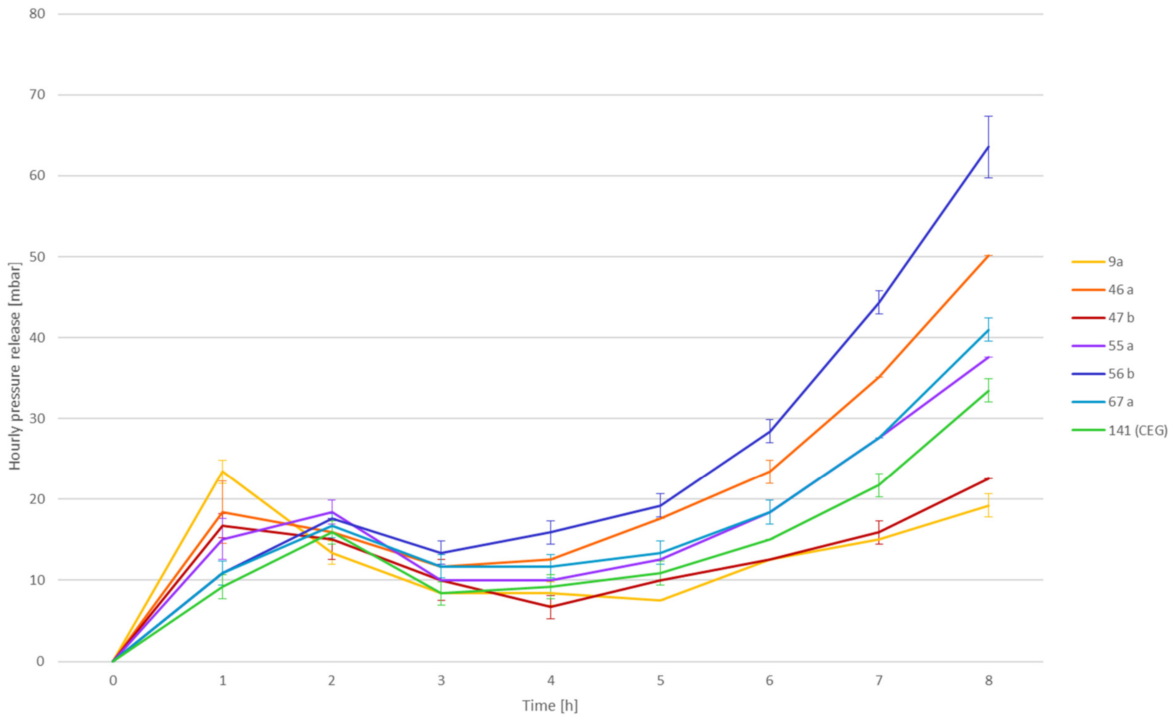

Figure S3. Triple fermentations with CEG and selected CEG spore clones of the first eight hours of fermentation. Ankom fermentations were monitored continuously to record CO2 pressure formation. Fermentations of the selected spore clones listed in Table 2 were run at 25°C. The parental CEG (#141) was used for comparison.

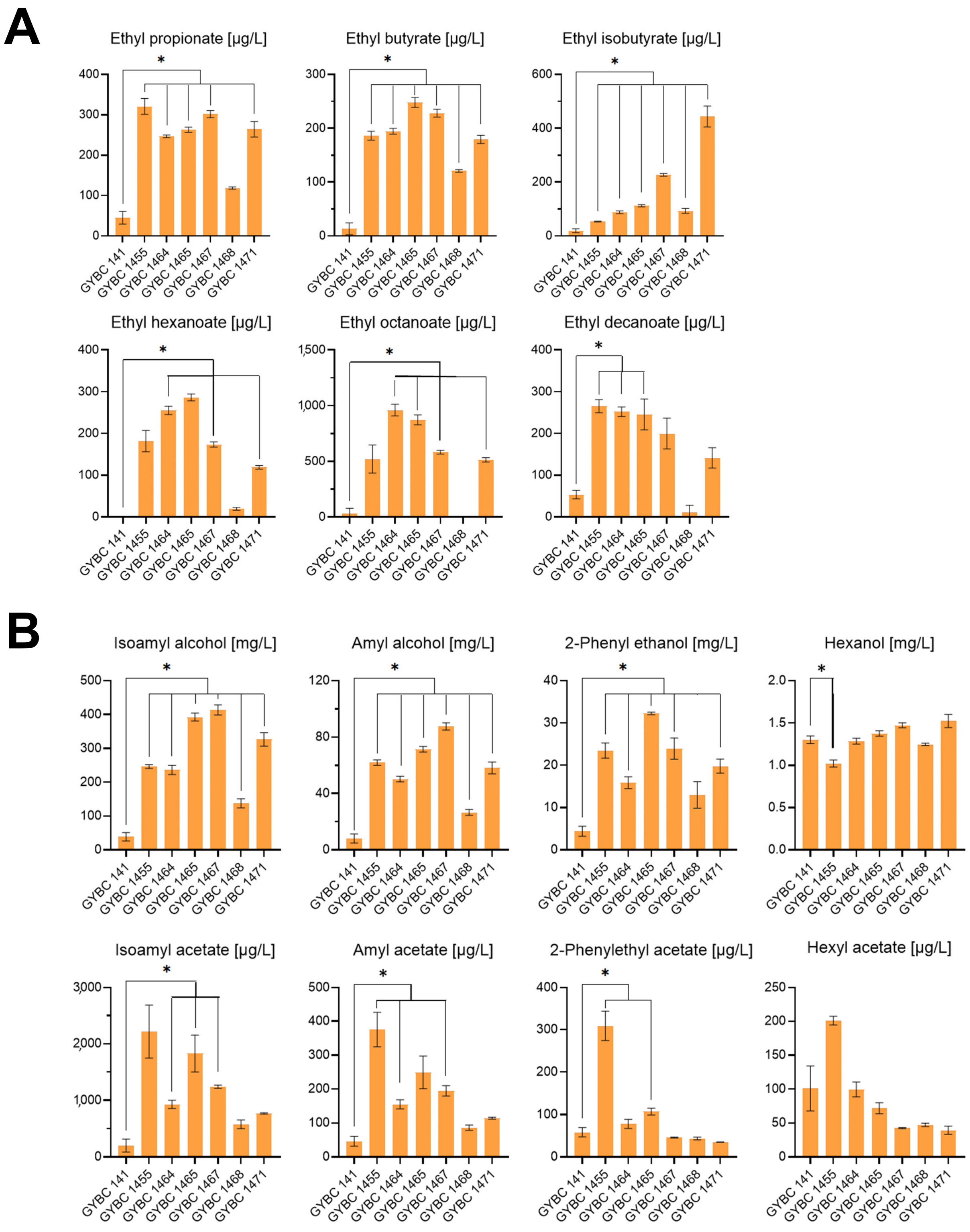

Figure S4. Aroma formation of selected CEG spore clones. *Amounts of **(A)** ethyl esters and **(B)** higher alcohols and their derived esters were quantified. Error bars indicate mean ± standard error of the mean. Asterisks indicate that CEG volatile aroma amounts were significantly different from the shown strains as determined by a two-tailed unpaired t-test with Welch's correction with* $p < 0.05$.

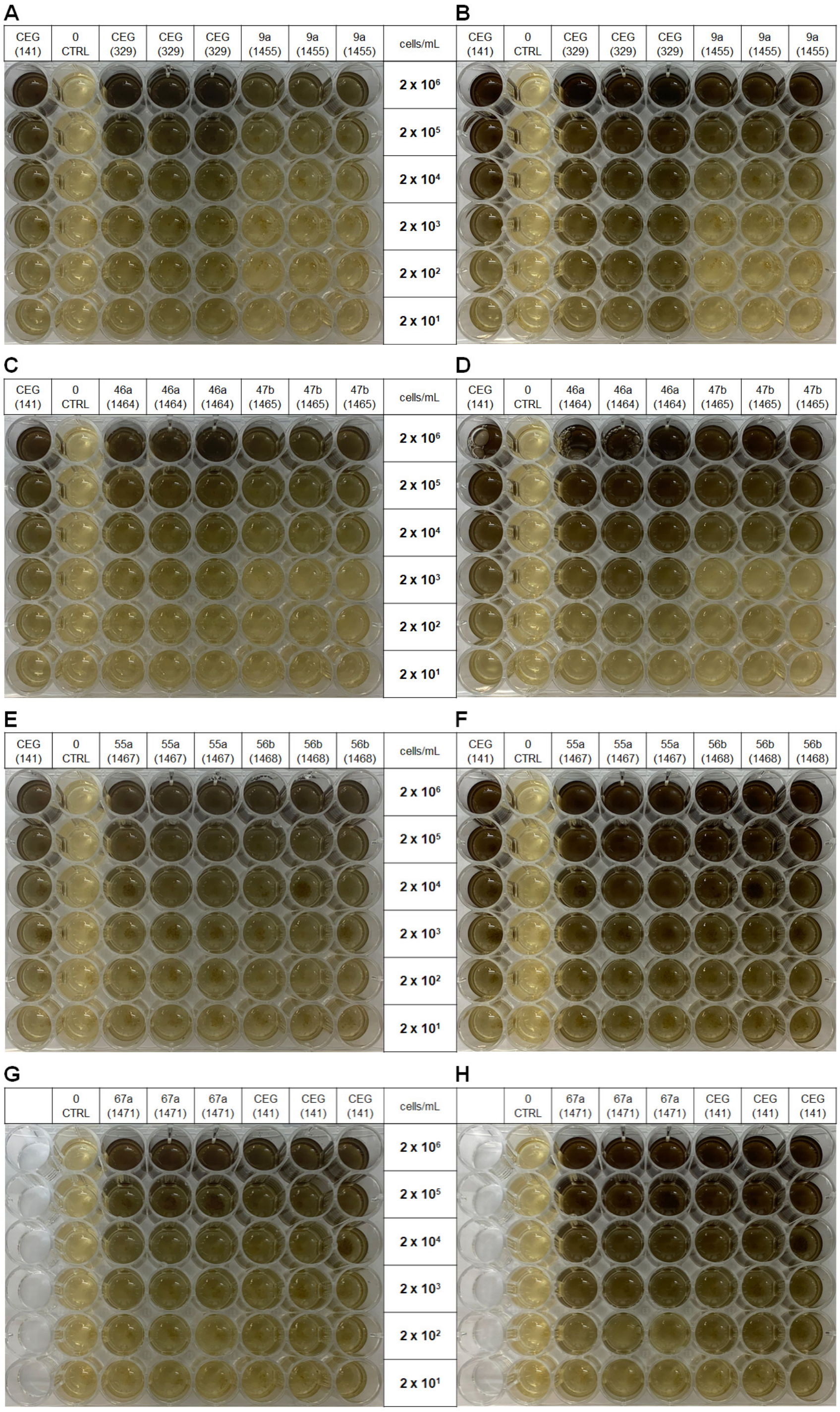

Figure S5. Qualitative assay for β-glucosidase activity. *Ten-fold serial dilutions of the indicated strains were prepared with esculin medium and incubated at 25°C. Images of the same plate were acquired after two days* ***(A,C,E,G)*** *and three days* ***(B,D,F,H)****. Darkness of wells correlates with -glucosidase activity.*

Table S1. Growth assays with CEG spore clones.

Nr.	Spore-Clone*	Growth in spot assay# 30°C	37°C	30°C 1M NaCl	10°C
1	1a	3	3	0	1
2	2a	3	2	1	0
3	3a	3	1	1	1
4	**4a**	3	0	0	1
5	4b	3	1	0	0
6	5a	3	1	0	0
7	6a	2	0	1	1
8	7a	2	0	0	0
9	8a	3	0	1	1
10	**9a**	3	3	0	1
11	**10a**	3	2	2	1
12	**11a**	2	1	1	1
13	**11b**	2	0	0	1
14	**12a**	3	3	1	1
15	**13a**	3	2	2	1
16	**14a**	3	2	2	1
17	**15a**	2	3	3	2
18	**16a**	2	1	1	1
19	17a	3	3	2	1
20	**17b**	3	2	2	1
21	18a	2	0	0	0
22	19a	3	2	1	1
23	20a	3	1	2	1
24	20b	3	1	1	1
25	21a	3	0	2	3
26	**22a**	3	3	2	2
27	23a	2	0	0	0
28	24a	3	1	0	0
29	25a	2	2	0	1
30	**25b**	3	2	2	1

Nr.	Spore-Clone	Growth in spot assay 30°C	37°C	30°C 1M NaCl	10°C
31	**26a**	3	2	2	1
32	27a	3	2	2	0
33	27b	2	2	2	0
34	28a	3	2	3	1
35	29a	3	1	1	0
36	29b	2	2	1	0
37	**29c**	2	0	1	0
38	30a	3	2	3	1
39	31a	3	0	1	0
40	31b	3	1	2	0
41	32a	3	1	2	0
42	32b	3	2	2	2
43	32,5a	2	0	0	0
44	33a	3	3	3	1
45	33b	2	0	0	0
46	34a	2	0	1	0
47	34b	2	0	0	0
48	35a	2	2	2	1
49	35,5a	3	3	3	1
50	**36a**	2	3	2	0
51	37a	2	2	0	1
52	37b	2	2	1	1
53	37,5a	2	1	1	1
54	37,5b	2	3	2	1
55	38a	3	1	1	1
56	**39a**	2	1	0	0
57	40a	2	1	0	1
58	40b	2	0	1	0
59	40c	3	2	3	2
60	41a	2	0	2	0

Nr.	Spore-Clone	Growth in spot assay 30°C	37°C	30°C 1M NaCl	10°C
61	**42a**	2	0	0	0
62	42,5a	2	2	1	1
63	43a	0,5	0	0	0
64	43b	3	2	1	0
65	43,5a	2	2	0	0
66	44a	2	2	2	0
67	45a	2	1	1	1
68	45b	2	1	0	0
69	**46a**	3	3	3	1
70	46b	2	2	1	0
71	47a	2	2	2	0
72	**47b**	2	3	1	1
73	**48a**	2	2	1	1
74	**48,5a**	2	2	0	1
75	**49a**	2	0	1	0
76	50a	1	0	0	1
77	50b	1	0	0	1
78	**51a**	2	3	2	2
79	51b	2	1	2	1
80	**51,5a**	3	2	1	1
81	52a	1	0	0	0
82	52b	3	1	1	1
83	53a	3	2	2	1
84	53b	3	2	2	1
85	54a	3	3	1	1
86	**55a**	3	3	2	1
87	56a	2	2	0	0
88	**56b**	3	3	3	2
89	57a	3	3	2	1
90	58a	3	3	2	1

Nr.	Spore-Clone	Growth in spot assay 30°C	37°C	30°C 1M NaCl	10°C
91	58b	2	1	0	0
92	**59a**	2	1	1	1
93	60a	2	2	1	1
94	61a	3	2	3	2
95	61b	3	2	2	2
96	**64a**	1	0	1	0
97	65a	2	1	2	1
98	66a	2	1	1	1
99	**67a**	2	3	2	1
100	**68a**	3	2	1	1
101	**69a**	2	2	1	1
102	69b	3	3	2	0
103	71a	2	1	1	0
104	73a	3	2	1	1
105	75a	3	1	2	1
106	76a	2	1	1	1
107	77a	2	1	0	0
108	77b	2	1	1	1
109	78a	3	1	1	1
110	79a	2	0	0	0
111	80a	2	1	1	2
112	**81a**	2	1	0	0
113	82a	2	1	0	1
141	CEG	3	2	1	1
142	GHM	3	3	2	1
804	**EC1118**	3	3	2	1
B 216	S. *kudriav-zevii*	2	0	1	2

*: spore clones that appear in bold print were tested positive for the FOT4 gene by PCR.

#: Growth of colonies was scored as very good (3), medium (2), reduced (1) or very low/no growth (0)

Table S2. HPLC dataset for single fermentations run at 25°C.

#	Glucose [g/L]	Fructose [g/L]	Total sugars [g/L]	Tartaric acid [g/L]	Malic acid [g/L]	Shikimic acid [mg/L]	Lactic acid [g/L]	Acetic acid [g/L]	Citric acid [g/L]	Ethanol [g/L]	Ethanol [%]
Must	85.1	84.0	169.2	5.6	4.8	39	<0,1	<0,1	0.19	1.5	0.2
GYBC 1452, 4a	0.0	3.3	3.3	5.6	2.6	42	nq	0.35	0.18	81.9	10.4
GYBC 1453, 6a	7.1	32.4	39.5	5.6	4.1	37	nq	0.24	0.17	62.6	7.9
GYBC 1454, 8a	40.6	63.7	104.3	5.8	4.3	38	nq	0.16	0.16	32.9	4.2
GYBC 1455, 9a	<1.0	<1.0	<1.0	6.0	3.9	36	nq	0.35	0.17	76.6	9.7
GYBC 1456, 11b	<1.0	<1.0	<1.0	5.9	3.4	37	nq	0.48	0.11	81.3	10.3
GYBC 1457, 12a	50.4	64.9	115.3	5.7	4.5	36	nq	<0,1	0.17	24.5	3.1
GYBC 1458, 15a	<1.0	<1.0	<1.0	5.8	3.5	35	nq	0.40	0.19	84.6	10.7
GYBC 1459, 21a	83.8	85.5	169.3	5.7	5.2	40	<0,1	<0,1	0.17	1.6	0.2
GYBC 1460, 22a	42.2	61.9	104.1	5.8	4.2	32	nq	0.22	0.10	30.8	3.9
GYBC 1461, 32b	41.6	63.3	104.9	5.5	4.0	36	nq	0.14	0.13	29.9	3.8
GYBC 1462, 36a	<1.0	<1.0	<1.0	5.7	3.7	33	nq	0.39	0.12	84.6	10.7
GYBC 1463, 40c	49.2	67.5	116.6	5.5	4.2	33	nq	0.08	0.15	24.5	3.1
GYBC 1464, 46a	<1.0	<1.0	<1.0	5.6	4.0	34	nq	0.55	0.11	81.2	10.3
GYBC 1465, 47b	<1.0	<1.0	<1.0	5.8	4.0	37	nq	0.53	0.13	81.9	10.4
GYBC 1466, 51a	1.2	14.0	15.1	5.5	3.5	32	nq	0.50	0.12	72.2	9.1
GYBC 1467, 55a	<1.0	<1.0	<1.0	5.5	4.3	35	nq	0.34	0.13	80.9	10.3
GYBC 1468, 56b	26.5	45.3	71.8	5.5	4.1	35	nq	0.27	0.09	43.6	5.5
GYBC 1469, 61a	44.1	61.8	105.9	5.6	4.5	31	nq	0.23	0.13	27.0	3.4
GYBC 1470, 61b	<1.0	<1.0	<1.0	5.5	3.7	36	nq	0.43	0.10	81.6	10.3
GYBC 1471, 67a	<1.0	<1.0	<1.0	5.3	4.0	35	nq	0.43	0.12	78.5	9.9
GYBC 1472, 80a	41.9	61.4	103.3	5.6	4.2	35	nq	0.26	0.12	28.3	3.6
CEG	37.9	58.6	96.5	5.5	4.4	35	nq	0.32	0.10	29.1	3.7

Strains that fermented both glucose and fructose completely are highlighted; nq: not quantifiable.

Table S3. HPLC dataset for single fermentations run at 10°C.

#	Glucose [g/L]	Fructose [g/L]	Total sugars [g/L]	Tartaric acid [g/L]	Malic acid [g/L]	Shikimic acid [mg/L]	Lactic acid [g/L]	Acetic acid [g/L]	Citric acid [g/L]	Ethanol [g/L]	Ethanol [%]
Must	80.3	78.2	158.6	5.6	4.6	38	<0.1	<0.1	0.14	1.9	0.2
GYBC 1452, 4a	13.2	42.3	55.5	5.6	3.9	37	<0.1	0.14	0.15	53.3	6.8
GYBC 1453, 6a	43.3	63.3	106.6	5.7	4.6	38	0.13	0.29	0.15	27.6	3.5
GYBC 1454, 8a	23.8	52.3	76.0	5.5	4.3	38	<0.1	0.20	0.15	42.5	5.4
GYBC 1455, 9a	39.2	57.4	96.6	5.6	4.5	34	<0.1	0.24	0.17	32.1	4.1
GYBC 1456, 11b	1.6	17.8	19.4	3.9	4.0	34	<0.1	0.22	0.15	70.1	8.9
GYBC 1457, 12a	13.3	41.2	54.5	5.6	4.2	37	0.12	0.36	0.13	52.7	6.7
GYBC 1458, 15a	40.5	53.1	93.6	5.6	4.4	34	<0.1	0.23	0.11	33.1	4.2
GYBC 1459, 21a	37.6	59.7	97.2	5.6	4.3	35	0.12	0.17	0.13	30.2	3.8
GYBC 1460, 22a	11.6	38.5	50.2	5.6	4.6	37	0.24	0.33	0.12	53.0	6.7
GYBC 1461, 32b	1.1	18.7	19.8	4.7	4.3	34	0.18	0.20	0.11	69.7	8.8
GYBC 1462, 36a	25.2	51.3	76.5	5.7	4.5	34	0.17	0.19	0.17	41.6	5.3
GYBC 1463, 40c	37.3	57.8	95.1	5.5	4.3	36	0.15	0.25	0.10	30.3	3.8
GYBC 1464, 46a	20.8	46.6	67.4	5.6	4.3	33	0.18	0.34	0.17	44.2	5.6
GYBC 1465, 47b	<1.0	<1.0	<1.0	5.3	4.6	36	0.15	0.25	0.18	80.9	10.3
GYBC 1466, 51a	<1.0	<1.0	<1.0	5.1	4.2	37	0.19	0.34	0.11	79.1	10.0
GYBC 1467, 55a	<1.0	<1.0	<1.0	5.8	4.5	35	0.15	0.30	0.12	80.1	10.1
GYBC 1468, 56b	<1.0	1.0	1.0	5.7	4.0	37	0.27	0.25	0.12	78.9	10.0
GYBC 1469, 61a	37.7	55.5	93.3	5.8	4.6	34	0.12	0.13	0.15	33.8	4.3
GYBC 1470, 61b	3.5	18.9	22.4	5.3	4.3	37	0.16	0.21	0.16	68.0	8.6
GYBC 1471, 67a	<1.0	<1.0	<1.0	5.4	4.3	34	0.24	0.46	0.10	79.2	10.0
GYBC 1472, 80a	6.7	28.7	35.4	5.7	4.2	36	0.28	0.22	0.16	60.4	7.7
CEG	24.2	48.1	72.3	5.6	4.2	34	0.22	0.14	0.12	40.8	5.2

Strains that fermented both glucose and fructose completely are highlighted.

Table S4. HPLC dataset for triplicate fermentations run at 25°C.

GYBC	Glucose [g/L]	Fructose [g/L]	Total sugar [g/L]	Acetic acid [g/L]	Ethanol [g/L]	Ethanol [%]
141	45.24 ± 4.64	60.54 ± 2.61	105.77 ± 7.22	0.25 ± 0.02	25.47 ± 3.37	3.20 ± 0.44
1455	<1.00*	<1.00*	<1.00*	0.40 ± 0.01*	78.13 ± 0.67*	9.90 ± 0.10*
1464	<1.00*	<1.00*	<1.00*	0.66 ± 0.02*	77.33 ± 0.46*	9.77 ± 0.06*
1465	<1.00*	<1.00*	<1.00*	0.47 ± 0.01*	78.03 ± 0.21*	9.90 ± 0.00*
1467	<1.00*	<1.00*	<1.00*	0.38 ± 0.02*	76.93 ± 1.27*	9.77 ± 0.15*
1468	25.82 ± 1.47	42.96 ± 1.16*	68.80 ± 2.62*	0.30 ± 0.03	44.50 ± 2.48*	5.63 ± 0.32*
1471	<1.00*	<1.00*	<1.00*	0.53 ± 0.01*	77.03 ± 0.50*	9.77 ± 0.06*

GYBC	Tartaric acid [g/L]	Malic acid [g/L]	Shikimic acid [mg/L]	Lactic acid [g/L]	Citric acid [g/L]
141	5.89 ± 0.04	4.95 ± 0.03	41.21 ± 1.78	0.16 ± 0.01	0.15 ± 0.00
1455	5.84 ± 0.02	4.20 ± 0.04*	43.77 ± 1.00	0.19 ± 0.01	0.10 ± 0.01*
1464	5.84 ± 0.03	4.12 ± 0.11*	41.80 ± 0.72	0.20 ± 0.03	0.12 ± 0.00
1465	5.88 ± 0.01	4.28 ± 0.07*	44.03 ± 0.69	0.16 ± 0.01	0.12 ± 0.00
1467	5.80 ± 0.10	4.31 ± 0.10*	45.01 ± 0.47	0.18 ± 0.02	0.10 ± 0.01*
1468	5.96 ± 0.10	4.44 ± 0.08*	44.43 ± 1.06	0.17 ± 0.01	0.14 ± 0.00
1471	5.87 ± 0.04	4.27 ± 0.03*	46.70 ± 0.52	0.40 ± 0.01*	0.10 ± 0.00*

Results are presented as the mean ± SD of three biological replicates. Significant differences in sugar degradation and ethanol formation as well as organic acid levels between the spore clones and their parental strain (GYBC 141) were determined using a classical ANOVA, including a homogeneity test with Welch´s correction and a Post Hoc test using the Games-Howell-test, $*p < 0.05$ as compared to the control.

Table S5. Maximum production rate of CO_2 ($m(CO_2)_{max}$ [Mol/h]) and the time of detection ($t_{m(CO2).max}$ [h]) of CEG and all spore clones during the fermentation in triplicates.

GYBC	$m(CO_2)_{max}$ [Mol/h]	$t_{m(CO2).max}$ [h]
141a	2.505E-05	16
141b	2.642E-05	17
141c	2.703E-05	17
1455a	3.902E-05	190
1455b	3.462E-05	190
1455c	3.963E-05	190
1464a	2.885E-05	190
1464b	4.828E-05	190
1464c	2.627E-05	190
1465a	2.232E-05	190
1465b	2.627E-05	190
1465c	2.050E-05	190
1467a	2.763E-05	21
1467b	2.930E-05	22
1467c	2.976E-05	23
1468a	3.750E-05	16
1468b	3.917E-05	17
1468c	3.902E-05	17
1471a	2.779E-05	24
1471b	2.870E-05	24
1471c	2.870E-05	24

Table S6. Quantification of aroma compounds.

GYBC	Isobutanol [mg/L]	Isoamyl alcohol [mg/L]	Amyl alcohol [mg/L]	2-Phenyl ethanol [mg/L]	Hexanol [mg/L]
141	58.04 ± 14.27	39.00 ± 12.40	8.08 ± 3.25	4.39 ± 1.20	1.30 ± 0.05
1455	180.82 ± 3.05*	246.76 ± 5.57*	61.97 ± 1.92*	23.47 ± 1.77*	1.02 ± 0.04*
1464	129.45 ± 5.36*	236.19 ± 13.45*	50.21 ± 2.00*	15.89 ± 1.40*	1.29 ± 0.04
1465	279.94 ± 11.72*	392.69 ± 11.75*	71.39 ± 1.99*	32.28 ± 0.30*	1.38 ± 0.03
1467	458.42 ± 8.91*	413.69 ± 14.67*	87.59 ± 2.52*	23.94 ± 2.53*	1.47 ± 0.03
1468	145.38 ± 8.31*	137.88 ± 13.31*	26.62 ± 2.12*	12.99 ± 3.14	1.25 ± 0.01
1471	515.43 ± 36.18*	326.73 ± 19.67*	58.17 ± 4.20*	19.81 ± 1.67*	1.53 ± 0.08

GYBC	Ethyl propionate [µg/L]	Ethyl butyrate [µg/L]	Ethyl isobutyrate [µg/L]	Ethyl hexanoate [µg/L]	Ethyl octanoate [µg/L]
141	45.28 ± 15.67	13.40 ± 11.00	19.48 ± 7.21	0.00 ± 0.00	31.21 ± 46.71
1455	320.83 ± 19.54*	186.23 ± 8.47*	53.57 ± 1.49*	181.51 ± 25.58	520.40 ± 126.12
1464	246.80 ± 3.37*	194.47 ± 5.38*	87.92 ± 4.97*	255.25 ± 10.01*	959.94 ± 51.89*
1465	263.03 ± 6.35*	248.17 ± 9.20*	112.53 ± 4.33*	286.30 ± 8.20	871.71 ± 43.40*
1467	302.01 ± 8.88*	228.20 ± 7.38*	227.00 ± 5.18*	173.46 ± 6.27*	582.53 ± 16.90*
1468	118.67 ± 2.91	120.77 ± 2.51*	93.19 ± 9.46*	19.15 ± 3.21	0.00 ± 0.00
1471	264.35 ± 19.11*	179.31 ± 7.53*	443.81 ± 39.03*	118.78 ± 4.29*	513.64 ± 19.94*

GYBC	Isoamyl acetate [µg/L]	Amyl acetate [µg/L]	Hexyl acetate [µg/L]	2-Phenylethyl acetate [µg/L]	Ethyl decanoate [µg/L]
141	202.16 ± 114.79	46.13 ± 14.53	101.06 ± 33.04	58.33 ± 10.99	53.49 ± 10.38
1455	2216.62 ± 469.94	375.21 ± 50.79*	200.84 ± 6.29	309.01 ± 34.62*	265.34 ± 15.82*
1464	928.78 ± 71.75*	155.08 ± 13.31*	99.63 ± 11.00	77.92 ± 10.60	252.05 ± 11.38*
1465	1829.53 ± 325.39*	249.29 ± 48.20	71.84 ± 8.25	107.19 ± 7.90*	245.47 ± 37.10*
1467	1242.06 ± 29.41*	194.96 ± 15.31*	42.55 ± 0.71	45.52 ± 0.97	199.65 ± 36.82
1468	577.98 ± 76.98	86.29 ± 7.80	47.37 ± 2.48	43.42 ± 3.15	11.50 ± 16.47
1471	769.61 ± 10.43	114.27 ± 2.82	39.40 ± 6.07	35.11 ± 0.54	141.21 ± 24.52

The results are shown as the mean ± SD of three biological replicates. Significant differences in the formation of higher alcohols, ethyl esters and acetate esters were determined between the spore clones and their parental strain (GYBC 141) using a classical ANOVA, including a homogeneity test with Welch´s correction and a Post Hoc test using the Games-Howell-test with *$p < 0.05$.

Table S7. Odor thresholds of Higher alcohols, acetate esters and ethyl esters, and their possible sensory effects on the aroma profile of wines.

Substance	Odor threshold in wine matrix [mg/L]	Possible sensory effects	
		Low quantities	High quantities
**Higher alcohols**			
Isobutanol	75.000 [38]	Clove-, fruity notes	Fusel-, spiritous notes
Isoamyl alcohol	30.000 [34]	Banana-, whiskey-, malt-, burnt-, fusel notes	harsh, nail polish notes
Amyl alcohol	211.800 [37]	Green notes	
2-Phenylethanol	14.000 [34]	Honey-, spice-, rose-, lilac notes	
Hexanol	4.000 [38]	Resin-, flower-, herbaceous-, green (cut grass) notes	
**Acetate esters**			
Isoamyl acetate	0.030 [34]	Banana-, pineapple notes	
Amyl acetate	0.313 [35]	Blackberry-, banana notes	
Hexyl acetate	0.670 [38]	Green-, pear-, apple-, cherry notes	
2-Phenylethyl acetate	0.250 [34]	Rose-, honey-, tobacco notes	
Ethyl acetate	7.500 [39]	Apple-, fruity notes	solvent notes
**Ethyl esters**			
Ethyl propionate	1.800 [40]	Fruity notes	
Ethyl butyrate	0.020 [34]	Apple-, pineapple-, tropical-, blueberry notes	
Ethyl isobutyrate	0.015 [34]	Sweet-, pineapple-, tropical notes	rubber notes

Ethyl hexanoate	0.014 [34]	Apple peel-, green apple-, tropical notes	
Ethyl octanoate	0.005 [34]	Pineapple-, tropical-, flower notes	Soapy notes
Ethyl decanoate	0.200 [34]	Grape-, brandy-, tropical notes	Soapy notes
Ethyl 2-hydroxy-4-methylvalerate	0.300 [36]	Fresh blackberry notes	
Diethyl succinate	1.200 [38]	Creamy-, full-bodied-, melon notes	
Ethyl lactate	150.000 [38]	Buttery-, creamy notes	

Table S8. Aroma formation [OAV]$^{\Delta}$ during the fermentations using CEG and its spore clones.

GYBC	Apple [OAV]	Pineapple [OAV]	Tropical [OAV]	Banana [OAV]	Rose/Honey [OAV]	Green [OAV]	Creamy/Full-bodied [OAV]	Fruity [OAV]
141	1.51 ± 1.04	14.95 ± 13.66	8.48 ± 9.89	8.19 ± 4.09	0.55 ± 0.12	0.51 ± 0.05	0.01 ± 0.01	19.54 ± 14.63
1455	31.29 ± 2.71*	190.85 ± 39.85*	131.25 ± 27.46*	83.31 ± 15.96*	2.91 ± 0.26*	0.85 ± 0.01*	0.16 ± 0.01*	230.64 ± 42.84*
1464	36.90 ± 0.98*	238.53 ± 8.61*	227.07 ± 10.67*	39.33 ± 2.73*	1.45 ± 0.14*	0.71 ± 0.01	0.18 ± 0.02*	280.47 ± 8.65*
1465	41.30 ± 0.34*	255.24 ± 6.94*	215.93 ± 8.67*	74.87 ± 10.98*	2.73 ± 0.03*	0.79 ± 0.02*	0.22 ± 0.01*	307.69 ± 6.62*
1467	30.49 ± 0.77*	184.45 ± 4.18*	156.44 ± 3.12*	55.82 ± 0.60*	1.89 ± 0.18*	0.85 ± 0.02*	0.22 ± 0.01*	232.70 ± 3.58*
1468	10.11 ± 0.61*	31.52 ± 3.19	13.68 ± 0.87	24.14 ± 3.03*	1.10 ± 0.24	0.51 ± 0.01	0.13 ± 0.01*	45.02 ± 4.58
1471	24.61 ± 0.91*	166.93 ± 6.19*	150.47 ± 6.26*	36.91 ± 1.01*	1.56 ± 0.12*	0.71 ± 0.04*	0.35 ± 0.02*	206.81 ± 8.12*

The results are shown as the mean ± SD of three biological replicates. Significant differences in the aroma formation was determined between the spore clones and their parental strain (GYBC 141) using a classical ANOVA, including a homogeneity test with Welch´s correction and a Post Hoc test using the Games-Howell-test with $^{*}p < 0.05$.

$^{\Delta}$ for OAV calculations [34-40] of descriptors the following categories and aroma compounds were used: **Pineapple flavor** was calculated from the OAV sum of isoamyl acetate, ethyl butyrate, ethyl isobutyrate, ethyl octanoate; **tropical flavor** based on ethyl hexanoate, ethyl decanoate, ethyl octanoate, ethyl butyrate, ethyl isobutyrate; **fruity flavor** as the most general descriptor delivered highest values and was calculated based on ethyl propionate, ethyl isobutyrate, ethyl butyrate, ethyl hexanoate, ethyl octanoate, ethyl decanoate, ethyl lactate, ethyl-2-hydroxy-4-methylvalerate, diethyl succinate, ethyl acetate, isoamyl acetate, amyl acetate, hexyl acetate, 2-phenylethyl acetate, isoamyl alcohol, amyl alcohol, hexanol, 2-phenylethanol, isobutanol; **apple** and **banana flavors** were calculated from hexyl acetate, ethyl hexanoate, ethyl acetate, ethyl butyrate and isoamyl alcohol, isoamyl acetate, amyl acetate, respectively; **honey/rose flavors** are derived from phenylalanine and include of 2-phenylethyl acetate, 2-phenylethanol; **green flavor** was based on hexanol, hexyl acetate, amyl alcohol; and **creamy/full bodied flavor** included ethyl lactate, diethyl succinate. Some aroma compounds appear in more than one category. Please note that OAVs do not directly indicate sensory quality because they do not consider matrix effects or interactions between compounds.

Table S9. The calculated odor active value [34-40] for the respective higher alcohols, acetate esters and ethyl esters.

**GYBC**	Isobutanol [OAV]	Isoamyl alcohol [OAV]	Amyl alcohol [OAV]	2-Phenyl ethanol [OAV]	Hexanol [OAV]
141	0.77 ± 0.19	1.30 ± 0.41	0.04 ± 0.02	0.31 ± 0.09	0.33 ± 0.01
1455	2.41 ± 0.04*	8.23 ± 0.19*	0.29 ± 0.01*	1.68 ± 0.13*	0.26 ± 0.01*
1464	1.73 ± 0.07*	7.87 ± 0.45*	0.24 ± 0.01*	1.13 ± 0.10*	0.32 ± 0.01
1465	3.73 ± 0.16*	13.09 ± 0.39*	0.34 ± 0.01*	2.31 ± 0.02*	0.34 ± 0.01
1467	6.11 ± 0.12*	13.79 ± 0.49*	0.41 ± 0.01*	1.71 ± 0.18*	0.37 ± 0.01*
1468	1.94 ± 0.11*	4.60 ± 0.44*	0.13 ± 0.01*	0.93 ± 0.22	0.31 ± 0.00
1471	6.87 ± 0.48*	10.89 ± 0.66*	0.27 ± 0.02*	1.42 ± 0.12*	0.38 ± 0.02

**GYBC**	Isoamyl acetate [OAV]	Amyl acetate [OAV]	Hexyl acetate [OAV]	2-Phenylethyl acetate [OAV]	Ethyl acetate [OAV]
141	6.74 ± 3.83	0.15 ± 0.05	0.15 ± 0.05	0.23 ± 0.04	0.69 ± 0.47
1455	73.89 ± 15.66	1.20 ± 0.16*	0.30 ± 0.01	1.24 ± 0.14*	8.71 ± 0.49*
1464	30.96 ± 2.39*	0.50 ± 0.04*	0.15 ± 0.02	0.31 ± 0.04	8.80 ± 0.17*
1465	60.98 ± 10.85*	0.80 ± 0.15	0.11 ± 0.01	0.43 ± 0.03*	8.33 ± 0.44*
1467	41.40 ± 0.98*	0.62 ± 0.05*	0.06 ± 0.00	0.18 ± 0.00	6.62 ± 0.09*
1468	19.27 ± 2.57	0.28 ± 0.02	0.07 ± 0.00	0.17 ± 0.01	2.63 ± 0.27*
1471	25.65 ± 0.35	0.37 ± 0.01	0.06 ± 0.01	0.14 ± 0.00	7.10 ± 0.30*

**GYBC**	Ethyl propionate [OAV]	Ethyl butyrate [OAV]	Ethyl isobutyrate [OAV]	Ethyl hexanoate [OAV]	Ethyl octanoate [OAV]

141	0.03 ± 0.01	0.67 ± 0.55	1.30 ± 0.48	0.00 ± 0.00	6.24 ± 9.34
1455	0.18 ± 0.01*	9.31 ± 0.42*	3.57 ± 0.10*	12.97 ± 1.83*	104.08 ± 25.22
1464	0.14 ± 0.00*	9.72 ± 0.27*	5.86 ± 0.33*	18.23 ± 0.72	191.99 ± 10.38*
1465	0.15 ± 0.00*	12.41 ± 0.46*	7.50 ± 0.29*	20.45 ± 0.59*	174.34 ± 8.68*
1467	0.17 ± 0.01*	11.41 ± 0.37*	15.13 ± 0.34*	12.39 ± 0.45*	116.51 ± 3.38*
1468	0.07 ± 0.00	6.04 ± 0.13*	6.21 ± 0.63*	1.37 ± 0.23*	0.00 ± 0.00
1471	0.15 ± 0.01*	8.97 ± 0.38*	29.59 ± 2.60*	8.48 ± 0.31*	102.73 ± 3.99*

**GYBC**	Ethyl decanoate [OAV]	Ethyl 2-hydroxy-4-methylvalerate [OAV]	Diethyl succinate [OAV]	Ethyl lactate [OAV]
141	0.27 ± 0.05	0.30 ± 0.06	0.00 ± 0.00	0.01 ± 0.01
1455	1.33 ± 0.08*	0.72 ± 0.22	0.09 ± 0.01*	0.06 ± 0.01*
1464	1.26 ± 0.06*	0.97 ± 0.29	0.11 ± 0.01	0.07 ± 0.02
1465	1.23 ± 0.19*	0.82 ± 0.11*	0.14 ± 0.01*	0.08 ± 0.00*
1467	1.00 ± 0.18	4.45 ± 0.19*	0.14 ± 0.01	0.08 ± 0.01*
1468	0.06 ± 0.08	0.78 ± 0.09*	0.00 ± 0.00	0.13 ± 0.01*
1471	0.71 ± 0.12	2.57 ± 0.11*	0.16 ± 0.01	0.20 ± 0.02*

The results are shown as the mean ± SD of three biological replicates. Significant differences in the aroma formation was determined between the spore clones and their parental strain (GYBC 141) using a classical ANOVA, including a homogeneity test with Welch´s correction and a Post Hoc test using the Games-Howell-test with $^{*}p < 0.05$. Please note that OAVs do not directly indicate sensory quality because they do not consider matrix effects or interactions between compounds.
